# Morphological Characteristics of Electrophysiologically Characterized Layer Vb Pyramidal Cells in Rat Barrel Cortex

**DOI:** 10.1371/journal.pone.0164004

**Published:** 2016-10-05

**Authors:** Jochen F. Staiger, Alexandre J. C. Loucif, Dirk Schubert, Martin Möck

**Affiliations:** 1 Institute for Neuroanatomy, University Medical Center, Georg-August-University, Göttingen, Germany; 2 Institute of Neuroanatomy, Albert-Ludwigs-University, Freiburg, Germany; 3 Donders Institute for Brain, Cognition & Behavior, Centre for Neuroscience, Radboud University Medical Centre, Nijmegen, The Netherlands; Georgia State University, UNITED STATES

## Abstract

Layer Vb pyramidal cells are the major output neurons of the neocortex and transmit the outcome of cortical columnar signal processing to distant target areas. At the same time they contribute to local tactile information processing by emitting recurrent axonal collaterals into the columnar microcircuitry. It is, however, not known how exactly the two types of pyramidal cells, called slender-tufted and thick-tufted, contribute to the local circuitry. Here, we investigated in the rat barrel cortex the detailed quantitative morphology of biocytin-filled layer Vb pyramidal cells in vitro, which were characterized for their intrinsic electrophysiology with special emphasis on their action potential firing pattern. Since we stained the same slices for cytochrome oxidase, we could also perform layer- and column-related analyses. Our results suggest that in layer Vb the unambiguous action potential firing patterns "regular spiking (RS)" and "repetitive burst spiking (RB)" (previously called intrinsically burst spiking) correlate well with a distinct morphology. RS pyramidal cells are somatodendritically of the slender-tufted type and possess numerous local intralaminar and intracolumnar axonal collaterals, mostly reaching layer I. By contrast, their transcolumnar projections are less well developed. The RB pyramidal cells are somatodendritically of the thick-tufted type and show only relatively sparse local axonal collaterals, which are preferentially emitted as long horizontal or oblique infragranular collaterals. However, contrary to many previous slice studies, a substantial number of these neurons also showed axonal collaterals reaching layer I. Thus, electrophysiologically defined pyramidal cells of layer Vb show an input and output pattern which suggests RS cells to be more "locally segregating" signal processors whereas RB cells seem to act more on a "global integrative" scale.

## Introduction

It is now commonly accepted that the neocortex is organized into an array of modules called functional columns which span across all layers [1]. Since its discovery by Woolsey and van der Loos [2], the primary somatosensory (barrel) cortex, has been extensively studied. This is because barrels in layer IV represent readily visualizable morphological correlates of functional columns with a defined sensory input [3,4]. The barrel cortex processes tactile information originating from the whiskers present on the contralateral snout of the animal [5,6]. Thus, the barrel cortex represents a prime model system to understand how cortical columns function [7–9]. Furthermore, an impressive quantitative data base now exists as an exquisite tool for neurocomputational approaches to cortical connectivity [10–12]. However, this database seems to be lacking information on layer Vb slender-tufted pyramidal cells (see Discussion).

Recently, the integration of sensory information at the cortical level has been extensively investigated (for reviews, see [13,14]). The major input station is layer IV, which receives information from different subdivisions of the thalamic ventroposterior medial nucleus [6]. Following principal whisker stimulation, the flow of excitation is strongly directed to the corresponding barrel-related column. Typically, the excitation reaches first layers IV and Vb, then layers II/III and finally layers Va and VI. With a lag of a few milliseconds, the excitation is intracortically propagated to neighboring columns [15]. This horizontal transmission of excitation is anatomically and functionally supposed to be preferentially mediated by long-range axonal projections of layer II/III and Vb pyramidal cells [16,17] but also by layer IV (star) pyramidal cells to a lesser extent [18–20].

Layer Vb pyramidal neurons have been subdivided into two major subclasses according to their electrophysiological properties [21–25], namely regular spiking (RS) cells and intrinsically burst spiking (IB) cells, the latter of which we call here repetitive burst spiking (RB; see Results for further explanation). These two types show also strongly correlated morphological features, which, however, never have been fully quantified. IB cells exhibit high-frequency bursts of action potentials in response to near-threshold depolarizing current stimulation. Morphologically, these cells show a large cell body, well-developed apical and basal dendrites and extensive horizontal axonal projections within infragranular layers (Va, Vb and VI). They project to the striatum and thalamus as well as other subcortical areas including tectum, brainstem and spinal cord [26–28]. RS cells display a regular spiking pattern in response to near-threshold depolarizing current stimulation. Morphologically, RS cells are different from IB cells, with smaller somata and more poorly developed apical as well as basal dendrites. The local axon collaterals of RS cells project vertically to supragranular layers (I and II/III) and, at least partially, lack projections to subcortical targets [26,27] but project ipsi- and contralaterally to the striatum [28,29]. However, only few quantitative descriptions of dendrites and axon arborizations were provided in barrel cortex [17,24,30,31]. Furthermore, with one exception [17], these studies did not examine the axonal and dendritic arborization patterns with respect to the septum and barrel-related columns. As functional columns are thought to be the basic unit of information processing, it is essential to know the distribution of dendritic and axonal arborizations with respect to the laminar and columnar compartments to better understand the integration of sensory information [9,13,32].

Layer Vb pyramidal cells receive inputs from all layers [8,24] and are the main output stage to other cortical areas, thalamus and brainstem [33,34]. However, RS and IB cells can be distinguished with respect to afferent inputs, both excitatory [24] and inhibitory [24,35–37]. They also qualitatively differ in their local connectivity [38], in their projection targets [28] as well as experience-dependent plasticity [39].

The present work aims at quantitatively investigating the morphology of electrophysiologically characterized layer Vb pyramidal cells in relation to barrel-related columns. Whole-cell patch clamp recordings of layer Vb cells were performed in combination with intracellular biocytin labeling in 300 μm-thick coronal slices containing the rat barrel cortex. We show that RS pyramidal cells are more column-restricted in their input as well as output domain than RB pyramidal cells, which extend both, dendrites and axons across the home barrel column.

## Materials and Methods

### Slice preparation

All experimental procedures were performed in accordance with German laws on animal research (TierSchG und TierSchVersV 2013). Most importantly, all steps were taken to ameliorate animal suffering. The experiments were approved by the Regierungspräsidium Freiburg and the animal care authorities of the University Freiburg. Male Wistar rats, aged 19 to 32 days, were used throughout the study. These animals were anaesthetized with isoflurane and subsequently decapitated. The brain was quickly removed and placed in an ice-cold artificial cerebrospinal fluid (ACSF), saturated with 95%O_2_-5%CO_2_. This ACSF contained (in mM): NaCl 124, KCl 3, NaH_2_PO_4_ 1.25, CaCl_2_ 1, MgCl_2_ 4 and glucose 10, buffered to pH 7.4 by NHCO_3_ (26 mM). Using a razor blade, two coronal cuts were carried out, one caudally to remove the cerebellum and one rostrally to remove the frontal part of the brain. The brain block obtained was glued to a metal platform of a HM650V vibratome (Microm). The vibratome chamber was then filled with the ice-cold ACSF and continuously bubbled with 95%O_2_-5%CO_2_. Coronal slices of 300 μm thickness containing the barrel cortex were obtained. The two hemispheres were separated with a cut through the midline. These slices were kept on a mesh in a storage chamber filled with ACSF and saturated with 95%O_2_-5%CO_2_ at room temperature (21–23°C).

### Electrophysiological recordings

The slices were transferred to a submerged chamber under an upright microscope (Axioskop FS, Zeiss, Germany). The recording chamber was continuously perfused at a flow rate of ~ 2 ml/min with a recording ACSF saturated with 95%O_2_-5%CO_2_ at 32°C. The recording ACSF contained (in mM): NaCl 124, KCl 3, NaH_2_PO_4_ 1.25, CaCl_2_ 1.6, MgCl_2_ 1.8 and glucose 10, buffered to pH 7.4 by NHCO_3_ (26 mM). The microscope was fitted with an infrared video camera (C5405-01, Hamamatsu Photonics, Japan), an image enhancer system (C2400, Hamamatsu Photonics, Japan) connected to a monochrome video monitor (WV-BM 1410, Panasonic). To select pyramidal cells located exclusively in barrel-related columns, the position of the barrel cortex and its layer Vb were identified at low magnification (4x) prior to each recording. Single pyramidal cells were then visually identified at higher magnification with a 40x water-immersion objective (Olympus). To document the position of the patch pipette in relation to the barrel-related column, the slices were photographed in the bath chamber after recordings.

Recording electrodes (5–6 MΩ resistance) were pulled from borosilicate glass tubes (Science Products, Hofheim, Germany) on a Narishige PP-830 puller (Narishige, Tokyo, Japan). The electrodes were filled with an internal solution containing (in mM): K-gluconate 117, KCl 13, CaCl_2_ 1, MgCl_2_ 2, K-HEPES 10, Na_2_ATP 2, NaGTP 0.5, EGTA 11, adjusted to pH 7.4 with KOH and with osmolarity set to 290–295 mOsm/l. Biocytin (0.5%) was added prior to each experiment.

Electrophysiological signals were acquired using an SEC-05L amplifier (npi-electronics, Germany). Membrane potentials were recorded in discontinuous current-clamp mode. Access resistance was monitored and compensated if changes appeared. Recordings during which the access resistance could not be compensated were discarded. Signals were low-pass filtered at 3 KHz and digitized at 20 KHz. Traces were monitored on a TDS2014 oscilloscope (Tectronix), acquired with a LIH 1600 interface (HEKA Electronik, Germany), using Tida 5.20 software (HEKA). All analyses of electrophysiological data were done with custom-made programs written in Signal5 script language (Cambridge Electronic Design, Cambridge, UK). All electrophysiological parameters were determined from recordings done at resting membrane potential. Data were not corrected for an estimated liquid junction potential of -10 mV.

The membrane potential baseline (V_m_) was calculated for each trace by averaging all data points prior to current pulse applications. The input resistance (R_in_) and membrane time constant (τ) were determined from averages of membrane potential responses to 10 consecutive square current pulses (-50 pA, 1 s duration). R_in_ was calculated according to Ohm’s law, τ was determined by fitting an exponential (f(x) = ae − ^x/b^ +c; a: amplitude, b: time constant, c: most negative membrane potential value). Because h-current was present in most of the cells, R_in_ and τ were determined for the maximal voltage response, i.e. prior to h-current activation. Additionally, we also calculated the steady-state input resistance (ssR_in_) for the last 20 ms of the voltage response before the current pulse was turn off. This allowed us (according to [40]) to estimate the strength of the h-current by calculating a sag index: sag index (%) = ((1/ ssR_in_− 1/R_in_)/ 1/ssR_in_) x 100.

Rheobase was measured by increasing the amplitude of clearly subthreshold square current pulses (500 ms duration) by 1 pA each step until firing threshold was reached. Firing threshold was estimated by a backwards search for the extrapolated membrane potential value corresponding to 10% of the slope of the upstroke. Action potential (AP) amplitude was calculated as the difference between firing threshold and AP peak. AP width is the duration of the AP at 50% of the amplitude. The maximal slope of APs is given by the maximum found in the 1^st^ derivative of the upstroke. All measurements of AP properties were done using rheobase stimulations and, as far as it concerns bursting neurons, the first AP of a burst. Inter-spike intervals were determined by calculating the period between the peaks of the first and the second AP in traces in which two APs were evoked at the lowest current injection amplitude.

The cells displayed either monophasic or compound afterpotentials. Afterhyperpolarizations (AHPs) were classified as fast (fAHP) or medium (mAHP), respectively. fAHPs reached their maximum within less than 5 ms whereas this lasts more than 10 ms for mAHPs (measured from where the AP downstroke crossed the firing threshold level). In the case of compound afterpotentials, the fAHP was usually separated from the mAHP by a short depolarizing hump. This hump was considered to be a true afterdepolarization only if its peak exceeded the firing threshold level. All amplitude measurements were done relative to the firing threshold level.

### Histochemical processing of biocytin

Slices were fixed in phosphate-buffered 4% paraformaldehyde for 24 h at 4°C. To visualize the biocytin-filled neurons, slices were processed as described previously [16,19]. Reconstruction of biocytin-labeled neurons selected for “completeness” were performed using a Nikon Eclipse 800 (Nikon, Ratingen, Germany) attached to a computer system (Neurolucida 9; MBF Bioscience). “Completeness” here means a soma position of at least 50 μm below the slice surface and an extension of the apical dendrite and axon parallel to the cutting plane. Thus, any truncation of the main stem of the apical dendrite or the axon (before it reached layer VIb) led to exclusion of the neurons from analysis. Data were not corrected for tissue shrinkage. The cells were reconstructed in relation to their barrel-related column using cytochrome oxidase staining [41] or the photomicrograph of the native slice for barrel identification. Then, the cells were quantitatively analyzed with Neuroexplorer (MBF Bioscience).

### Statistical analysis

Statistical analysis was performed using Mann-Whitney rank sum tests followed by post hoc Bonferroni corrections (SigmaPlot12; Systat Software Inc., San Jose, CA, USA). Data are presented as mean ± SD.

## Results

### Electrophysiological characterization of layer Vb pyramidal cells

#### Action potential firing patterns

**Fig 1:**
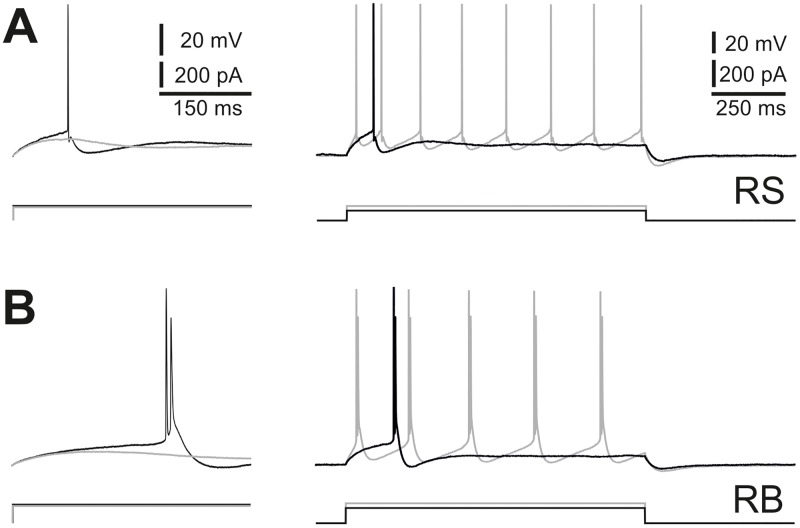
Classification of firing pattern types of layer Vb pyramidal cells. (A, D) Representative examples for the 2 major firing pattern types observed in our sample. Voltage responses to rheobase stimulations (black) and just subthreshold (rheobase minus 1 pA) stimulations (gray) are shown in higher temporal resolution in the left panels. The entire voltage response to rheobase (black) and rheobase plus 50 pA (gray) stimulations are shown in the right panels. Voltage traces are always displayed above the corresponding rectangular current traces. **(A)** Regular spiking (RS) neurons fired a single action potential (AP) if depolarized just above threshold. Clearly, suprathreshold current pulses evoked trains of single APs with weak firing rate adaptation. **(B)** Repetitive burst spiking (RB) neurons fired one or several AP doublets in response to rheobase or clearly suprathreshold stimulations, respectively.

All recorded neurons (n = 318) showed one out of four action potential (AP) firing patterns that are known to be typical for cortical excitatory neurons: (i) regular spiking (RS; n = 204) and (ii) repetitive burst spiking (RB; n = 67), which represented 85% of our sample as well as (iii) high-threshold bursting (HTB; n = 7) and (iv) initial doublet (ID; n = 40), which were found in only 15% of all cases (Fig 1 and S1 Fig). In this study we focused on the majority population RS and RB, for which we also compiled a sufficient number of well-filled neurons for morphological reconstructions (see below). RS cells, with or without a depolarizing afterpotential, always fired single APs at rheobase and trains of slightly adapting APs at stronger suprathreshold stimulation (Fig 1A). RB cells showed bursts consisting of doublets already at rheobase as well as any stronger suprathreshold stimulus strengths (Fig 1B). In the present sample, only a single RB cell displayed bursts that consisted of 3 APs.

These data suggest that layer Vb pyramidal cells, depending on their intrinsic firing properties, can send differently coded messages to their downstream targets [42,43].

#### Basic electrophysiological properties of RS and RB pyramidal cells

To show their viability and to make them comparable to previous reports, we characterized basic passive and active electrophysiological properties (Table 1).

**Table 1:** Basic subthreshold and suprathreshold electrophysiological properties of RS versus RB pyramidal cells.

	RS cells	RB cells	P value
**V**_**m**_ **[mV]**	-59.8±3.3	-61.7±1.9	>0.05
**R**_**in**_ **[M**Ω**]**	82.7±40.7	46.8±18.6	>0.05
**τ [ms]**	22.6±7.1	16.2±5.1	>0.05
**sag index [%]**	38.8±9.5	36.6±9.2	>0.05
**rheobase [pA]**	69.9±46.1	126.1±64.9	>0.05
**1. AP threshold [mV]**	-42.6±4.6	-48.4±3.8	>0.05
**1. AP width [ms]**	1.1±0.2	1.2±0.2	>0.05
**1. AP amplitude [mV]**	81.0±6.5	87.4±6.1	>0.05
**fAHP amplitude] mV]**	-6.7±1.9	-	-
**fAHP time to peak [ms]**	1.1±0.3	-	-
**mAHP amplitude [mV]**	-15.3±2.4	-18.6±3.8	>0.05
**mAHP time to peak [ms]**	42.4±7.0	57.2±13.9	>0.05
**1. ISI [ms]**	164.3±40.3	5.8±0.6	<0.001

Abbreviations: AP—action potential; fAHP—fast afterhyperpolarization; ISI—inter-spike interval; mAHP—medium afterhyperpolarization; R_in_−input resistance; **τ** –membrane time constant; V_m_−resting membrane potential.

The somatic V_m_, measured immediately after establishing whole cell configuration, was close to -60 mV for both cell types. As expected for such large neurons, R_in_ was comparably low, with means below 100 MΩ for both RS and RB cells. However, the average R_in_ of RS cells was almost twice as large as the one of RB cells. We also observed a substantial difference in the membrane time constants between the two cell types, **τ** on average being ~40% longer for RS cells than for RB cells (22.6 ± 7.1 ms and 16.2 ± 5.1 ms, respectively). In response to hyperpolarizing current pulses both types of pyramidal cells showed delayed voltage sags and rebound depolarizations (data not shown) indicating the presence of h-current. In both cases the initial hyperpolarization was on average decreased by more than one-third. As listed in Table 1, none of the subthreshold membrane properties (R_in_, **τ**, V_m,_ and sag index), however, showed significant differences between RS and RB cells.

As mentioned before, RB cells fired a doublet in response to just suprathreshold current pulses whereas RS cells fired only a single AP. The rheobase tended to be larger for RB cells, however, we observed a considerable inter-individual variability (RB: 126.1 ± 64.9 pA; RS: 69.9 ± 46.1 pA). Accordingly, the two groups did not differ significantly in rheobase. The mean firing threshold of the first AP in a doublet in RB cells was almost 6 mV lower than that of RS cells (-48.4 ± 3.8 mV and -42.6 ± 4.6 mV, respectively). Again, due to variability within the two groups, we observed no statistical difference. Because the two firing patterns are qualitatively very different, a burst of two APs with no AHP in between but followed by a monophasic mAHP versus a single AP followed by variable AHP sequences, it is difficult to compare the waveforms directly. In terms of amplitude and width at half-amplitude of the first AP the two cell types were statistically indistinguishable. RB cells invariably showed only mAHPs (Fig 1B, left panel) of considerable amplitude (-18.6 ± 3.8 mV) and slow kinetics (time to peak: 57.2 ± 13.9 ms). mAHPs were also present in all RS cells but in 55% of the cases they were preceded by a fAHP (Fig 1A, left panel). The mAHP amplitudes in RS cells (-15.3 ± 2.4 mV) was not statistically different from the ones in RB cells (-18.6 ± 3.8 mV) and they did not peak significantly faster (42.4 ± 7.0 ms versus 57.2 ± 13.9ms). fAHPs were much smaller in amplitude (-6.7 ± 1.9 mV) and much faster (time to peak: 1.1 ± 0.3 ms).

### Morphological characterization of layer Vb pyramidal cells

#### Somatodendritic properties

**Fig 2:**
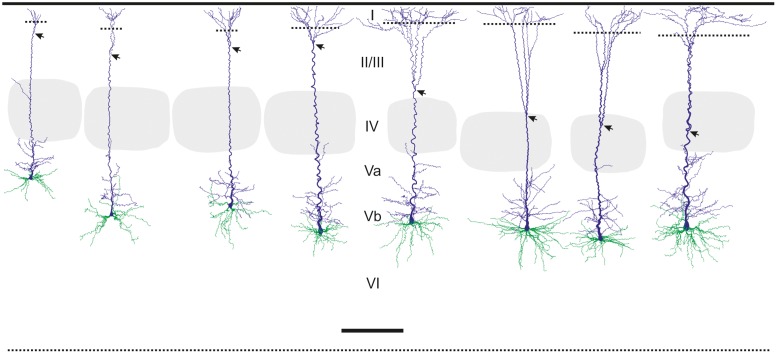
Somatodendritic reconstructions of regular spiking (RS) pyramidal cells in comparison to repetitive burst spiking (RB) neurons of layer Vb. Roman numerals in the middle indicate cortical layers. Stippled lines demarcate the easily identifiable border of layer I and layer II/III. To the left, 4 examples of RS neurons (the left most coming from layer Va to illustrate their similarity) are shown whereas to the right 4 examples of RB cells are depicted, all coming from a comparable location within layer Vb. The arrow indicates the position on the apical dendrite where the main stem starts to bifurcate in a regular dichotomous manner. Please note that this happens within the top or middle of layer II/III for RS cells whereas it occurs close to or even within layer IV for RB cells. The respective home barrel defining the columnar boundaries is depicted in light gray for each neuron. The soma and the apical dendrite are colored in blue, the basal dendrites in green. Scale bar, 300 μm.

RS (n = 16) and RB (n = 17) pyramidal cells were well-filled and sufficiently completely recovered (i.e. no first or second order branches severed). They resembled classical pyramidal neurons, showing an ovoid to pyramidal shaped soma from which one apical dendrite (directed toward the pial surface) and several basal dendrites (directed laterally or toward the white matter) emanated. The basal dendrites immediately ramified in the typical dichotomous fashion whereas the apical dendrite extended a main stem from which thinner oblique side branches emerged until a certain point at which it started to branch dichotomously as well, to form the classical terminal tuft. The spot from which this branching into the terminal tuft starts is significantly deeper in RB (414.44 ± 109.45 μm below pia) than in RS (252.31 ± 76.90 μm below pia) cells (p<0.001; Fig 2). Interestingly both, RS and RB cells possessed two main locations where dendritic arborization was densest: (i) the terminal tuft, exclusively formed by the apical dendrite in layers I and II/III and (ii) a perisomatic “tuft” that is jointly formed by all basal dendrites and the numerous proximal oblique side branches of the apical dendrite. This perisomatic dendritic sphere extended from layer Vb mainly into neighboring layer Va (for detailed quantification see S1 Table).

At a qualitative level, RS pyramidal cells have the appearance of the slender-tufted type whereas RB cells resemble thick-tufted pyramidal cells [17,44]. This was reflected by many of the quantified properties. RB cells have significantly larger somata than RS cells (soma area 430.2±85.53 versus 319.0±79.1 μm^2^; p = 0.005). Also the thickness of the dendrites and their overall length was greater in RB than in RS cells. This holds true for basal as well as apical dendrites (Table 2 and S1 Table).

**Table 2:** Basic somatodendritic properties of RS versus RB pyramidal cells.

	RS cells	RB cells	P value
**Feret max (μm)**	24.9±3.3	31.4±3.2	<0.001
**Feret min (μm)**	17.1±2.8	19.9±2.1	0.023
**Soma area (μm**^**2**^**)**	319.00±79.06	430.24±85.70	0.005
**Dendrites (n)**	6.2±1.5	6.5±0.8	>0.05
**AD thickness (μm)**	4.58±0.84	6.43±0.98	<0.001
**AD ends (n)**	40.6±10.4	48.6±8.7	>0.05
**AD branch point depth (μm below pia)**	252.31±76.90	414.44±109.45	<0.001
**BD maximal thickness (μm)**	3.03±0.68	4.02±0.73	<0.001
**BD ends (n)**	24.6±5.3	37.2±7.6	<0.001
**AD length (μm)**	6706.4±1710.5	8596.4±1387.1	0.020
**BD length (μm)**	3060.4±809.3	5479.9±1408.6	<0.001
**Dendritic span-vertical (μm)**	1141.5±137.7	1122.5±90.1	>0.05
**Dendritic span-horizontal (BD tuft) (μm)**	267.9±56.9	369.3±52.1	<0.001
**Dendritic span-horizontal (AD tuft) (μm)**	248.4±68.0	447.6±93.4	<0.001

Abbreviations: AD—apical dendrite; BD—basal dendrite; for a full characterization, including layers and columns, see S1 Table.

**Fig 3:**
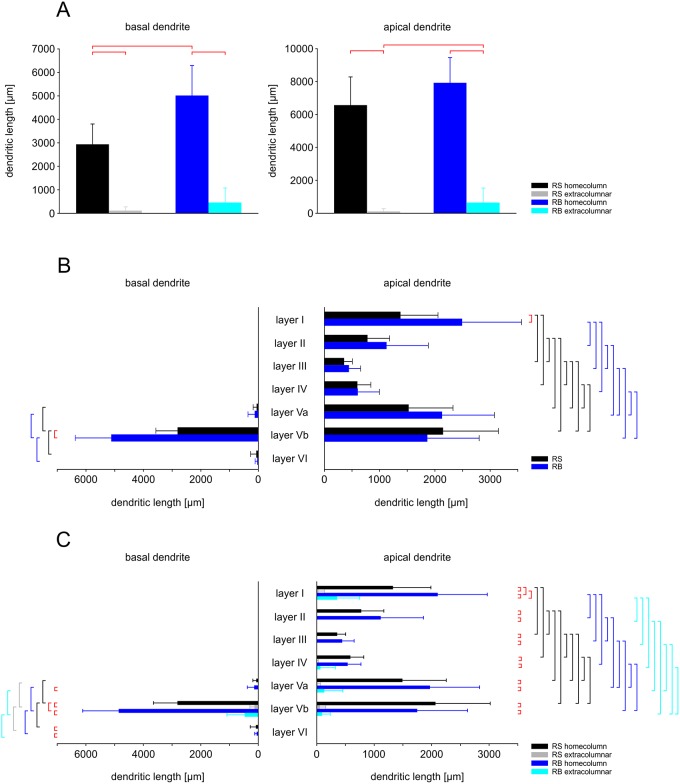
Distribution of basal and apical dendritic length across layers and columns in RS versus RB pyramidal cells. **(A)** Layer-independent, absolute number of basal and apical dendritic length in the home column and outside of it (extracolumnar) for both RS and RB (see color codes on the right hand side). As indicted by the red horizontal bars at the top, RB cells have significantly longer basal dendrites in the home column as RS cells. A similar difference exists for the apical dendrite outside the home column. In both cell types, however, dendrites are significantly more confined to the home column as compared to neighboring compartments. (**B**) The absolute length of basal and apical dendrites, independent of column borders, shows layer-dependent differences for both RS and RB cells. Vertical bars on both sides of the plots indicate significant differences (colors corresponding to the color code on the right hand side or red for differences between RS and RB). In both cell types, layer Vb houses most of the basal dendrites, in RB even more than RS. Layers I, Va, and Vb house most of the apical dendrite. The apical dendritic length in the remaining layers is significantly smaller. The only difference between RS and RB occurs in layer I. **(C)** Absolute length of basal and apical dendrites with respect to column borders and layers for both RS and RB. Significant differences are indicated as in (B). In both cell types there is significantly more basal dendrite in layer Vb than in Va and VI. This holds true for home column and neighboring compartments. Within layer Vb home column, RB cells have more basal dendrite than RS cells. With the exception of layer Va in RS cells, the home column compartments always contained more basal dendrite than the corresponding neighboring compartments. As in (B) layers I, Va, and Vb house more apical dendrite as the remaining ones in both, RS and RB. In both cell types there is more apical dendrite within the home column as compared to neighboring compartments. Again, the only significant difference between RS and RB occurs in layer I with RB cells having more apical dendrite in both the home column and outside of it. For mean ± standard deviation of the values plotted in this Fig, see S3 Table.

Since the dendritic trees, as the input compartment, were distributed over many different layers, which receive specific afferent projections [45], we analyzed them in more detail. The absolute layer-specific length of dendrites in the home column for RS cells was: basal dendrite LVa 57.81 ± 124.68 μm (on average corresponding to a relative proportion of 1.97%), LVb 2803.13 ± 837.32 μm (95.56%) and LVI 72.38 ± 202.51 μm (2.47%); apical dendrite LI 1327.44 ± 658.55 μm (20.22%), LII 774 ± 393.25 μm (11.79%), LIII 355.94 ± 146.92 (5.42%), LIV 584.87 ± 233.33 μm (8.35%), LVa 1491.75 ± 762.26 μm (22.73%) and LVb 2066.88 ± 952.15 (31.49%). For RB cells, the layer-specific length of dendrites was as follows: basal dendrite LVa 126.10 ± 241.37 μm (2.52%), LVb 4847.94 ± 1260.71 μm (96.71%) and LVI 37.88 ± 47.88 μm (0.77%); apical dendrite LI 2106.53 ± 861.70 μm (26.60%), LII 1113.53 ± 743.70 μm (14.06%), LIII 442.35 ± 207.11 μm (5.59%), LIV 537.41 ± 231.71 μm (6.79%), LVa 1970.82 ± 863.61 μm (24.89%) and LVb 1748.59 ± 875.90 μm (22.06%). Thus, the two hot spots in terms of densest dendritic arborization in the home column are for apical dendrites layers I (20.22% in RS and 22.60% in RB) and Va (24.89% in RB) or Vb (31.49% in RS), whereas for the basal dendrites there is a single hot spot in layer Vb (95.56% in RS and 96.71% in RB). The dendritic length was much larger in the home column than outside of it for basal as well as apical dendrites in RB compared to RS cells. However, RB cells distributed significantly more apical dendrite in these “extra”-home column (i.e. septum and neighboring column) compartments. These differences are visualized in the histograms of Fig 3 and S2 Fig.

Thus, these data clearly indicate that RB cells can integrate much more numerous inputs over a wider spatial domain than RS cells.

#### Axonal properties

**Fig 4:**
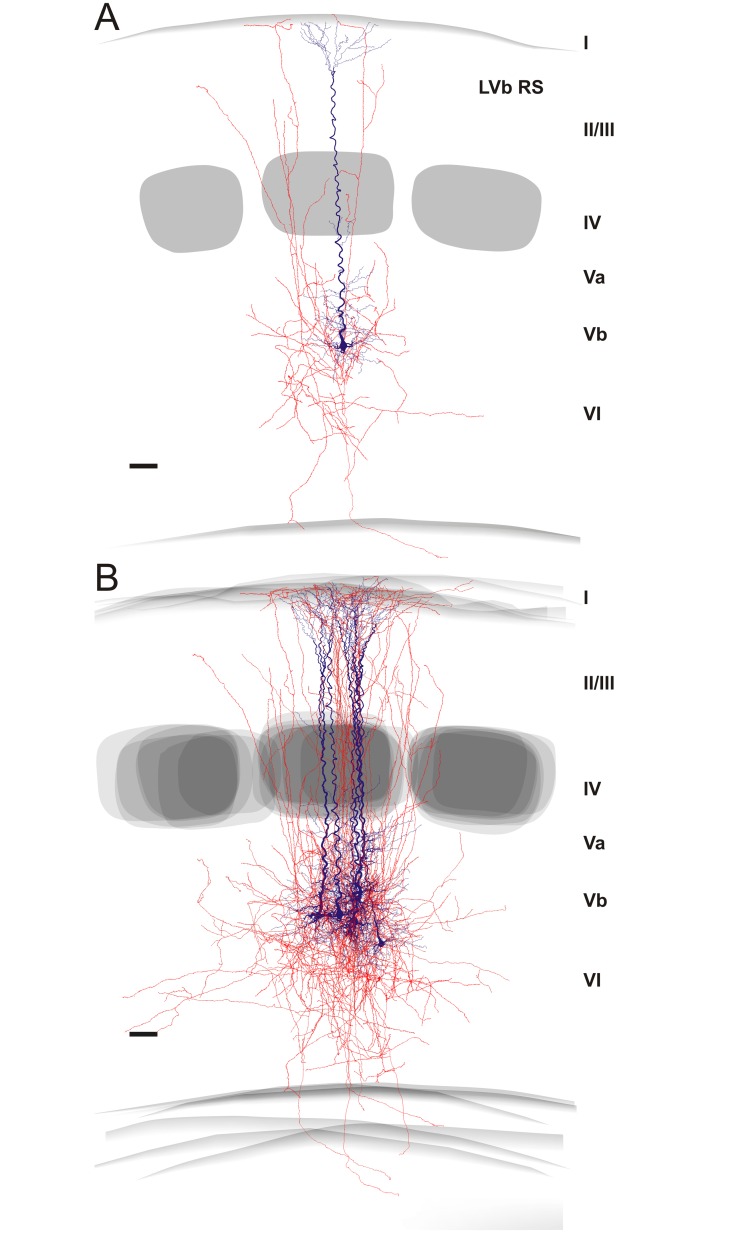
Reconstructions of regular spiking (RS) pyramidal cells. **(A)** Individual example of a well-preserved neuron. **(B)** Overlay of 6 neurons, aligned with respect to their home barrel. Roman numerals indicate cortical layers. Soma and dendrites in blue, axons in red. Barrels are shown as gray layer IV objects. Please note the locally very dense axonal arbor and the recurrent collaterals, which seem to target layer I. Scale bar, 300 μm.

**Fig 5:**
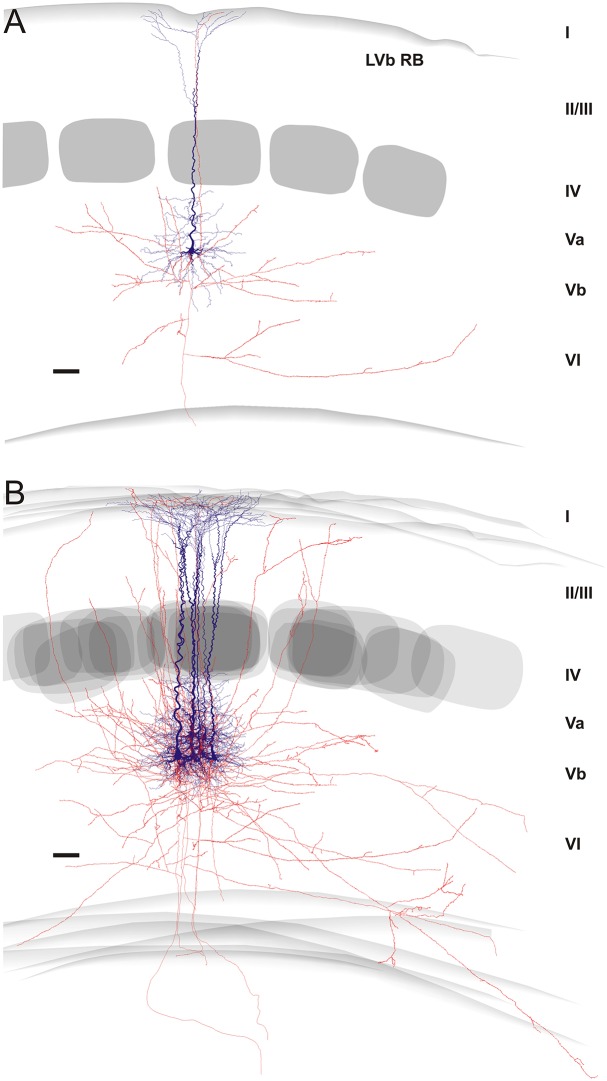
Reconstructions of repetitive burst spiking (RB) pyramidal cells. **(A)** Individual example of a well-preserved neuron. **(B)** Overlay of 6 neurons, aligned with respect to their home barrel. Roman numerals indicate cortical layers. Soma and dendrites in blue, axons in red. Barrels are shown as gray layer IV objects. Please note that axonal arbors are locally very sparse and the overall picture is dominated by horizontal collaterals, which seem to target the neighboring columns. Scale bar, 300 μm.

Both, RS (n = 12) and RB (n = 13) pyramidal cells issue an axonal main stem from the basal part of the soma that was invariably directed toward the white matter. From this main stem numerous ascending, oblique or horizontal intracortical collaterals budded off, before it reached the subcortical white matter. At the level of individual, well-preserved neurons, it is obvious that all layers of the home column are supplied with at least one collateral (Figs 4A and 5A). The density of boutons per 100 μm axon was similar for both types (22.4 ± 4.5 for RS versus 20.4 ± 1.9 for RB; p>0.05) and, accordingly, the longer total axonal length of RS cells comes with a higher number of boutons in comparison to RB cells (Table 3). The axonal span mimics the thickness of the cortex in the vertical direction whereas in the horizontal direction, RB cells had a significantly wider spread than RS cells (Table 3). However, all LVb pyramidal cells were able to target at least first (RS cells) and second order (RB cells) neighboring barrel-related columns.

**Table 3:** Basic axonal properties of RS versus RB pyramidal cells.

	RS cells	RB cells	P value
**Length (μm)**	15903.6±5336.3	9596.6±3586.1	0.025
**Boutons (n)**	3572.8±1583.3	1821.3±830.5	0.049
**Density (n/100 μm)**	22.4±4.5	20.4±1.9	>0.05
**Nodes (n)**	67.8±24.6	39.8±21.6	0.045
**Axonal span-horizontal (μm)**	1018.3±477.8	1393.6±337.0	0.049

For a full characterization, including layers and columns, see S2 Table.

Certainly, severing of axons at the slice surface is an issue for each individual neuron of such a huge size, but due to careful selection of only well-filled and least-truncated neurons for reconstruction, consistent features in the intracortical connectivity (reflected by boutons, which are considered to be presynaptic specializations) differentiating RS from RB cells became obvious (Figs 3 and 4). In RS pyramidal cells 81.2% of the axonal boutons were distributed within the home column and accordingly only 18.8% extended into adjacent septal compartments or neighboring columns (p <0.001). In comparison, only 52.1% of the axon stayed in the home column and 47.9% extended into the neighboring columns for RB cells (p >0.05). This means that RS cells retain significantly more axon in their home column than RB cell (p <0.001). Interestingly, the relative distribution of axon across the different layers (see below) was similar for nearly all columnar compartments (home, septal, neighboring, the latter two of which are pooled in the statistical analyses as “extra”-home column).

**Fig 6:**
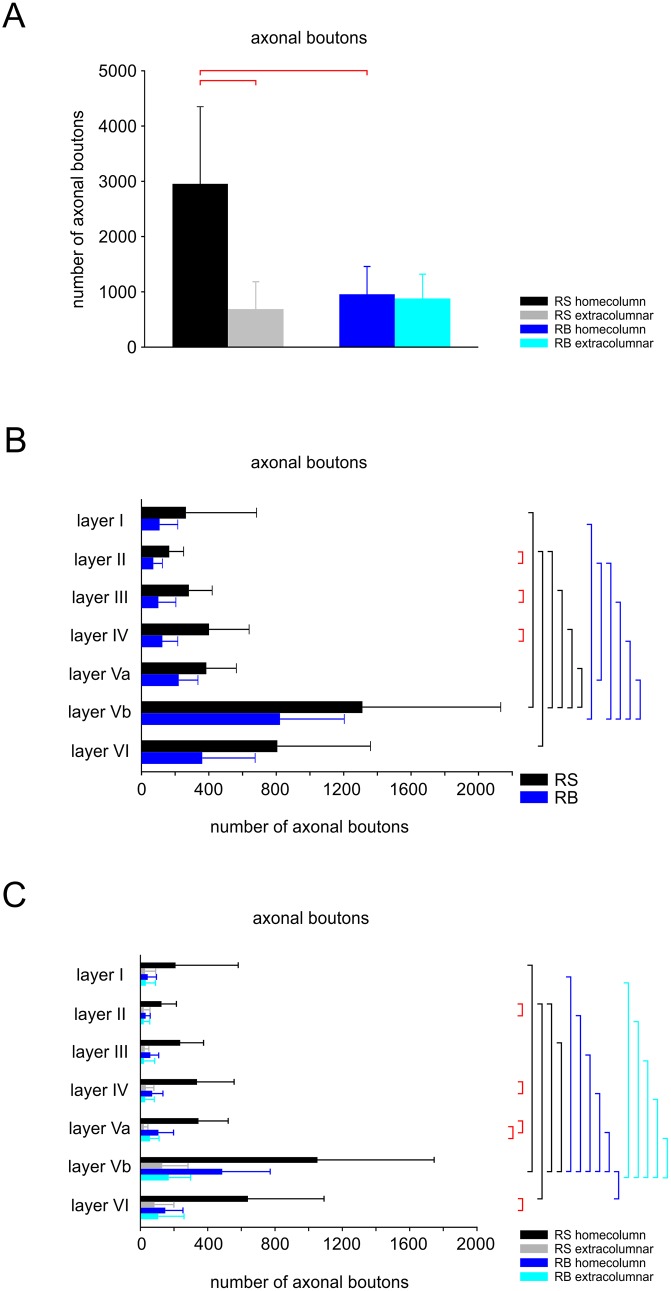
Distribution of axonal bouton number across layers and columns. **(A)** Total number of axonal boutons across all layers in the home column and in neighboring compartments (extracolumnar) for both, RS and RB cells (see color codes on the right hand side). As indicated by the red horizontal bars at the top, the axons of RS form significantly more boutons in their home column than outside of it. There are also significantly more boutons in RS as compared to RB in home column. RB cells form indistinguishable numbers of axonal boutons in their home column and in neighboring compartments. **(B)** Layer-dependent, absolute number of axonal boutons for RS and RB cells (independent of column borders). Vertical bars on the right hand side indicate significant differences (colors corresponding to the color code below these bars or red for differences between RS and RB cells). In both cell types there are significantly more axonal boutons in layer Vb than in other layers except for layer VI. Significant differences between RS and RB occur only in layers II to IV. **(C)** Absolute numbers of axonal boutons with respect to column borders and layers for RS and RB. Significant differences are indicated as in (B). In RS and RB, layer Vb home column houses more axonal boutons than any other layer in the home column except for layer VI in RS. While there is no layer-dependent difference in the number of boutons outside the home column in RS, RB cells have significantly more axonal boutons outside the home column in layer Vb than in other layers, except for layer VI. Differences between RS and RB occur in layers II, IV, Va, and VI in home column and in layer Va in neighboring compartments. For mean ± standard deviation of the values plotted in this figure, see S4 Table.

Within the home column, the axonal bouton number was allocated for RS cells to LI 209.58 ± 371.94 (on average corresponding to 7.1%), LII 125.92 ± 88.39 (4.27%), LIII 236.75 ± 139.00 (8.03%), LIV 337.00 ± 220.05 (11.4%), LVa 345.75 ± 176.45 (11.7%), LVb 1051.67 ± 693.13 (37.7%) and LVI 639.75 ± 452.03 (21.7%). In RB cells, these numbers amounted to 44.23 ± 51.73 (4.7%) in LI, 31.62 ± 27.33 (3.33%) in LII, 59.08 ± 50.14 (6.23%) in LIII, 69.77 ± 63.98 (7.7%) in LIV, 107.77 ± 89.76 (11.4%) in LVa, 487.23 ± 284.14 (51.4%) in LVb and 148.69 ± 104.42 (15.7%) in LVI. These values were significantly different between the two cell types in LII, LIV, LVa and LVI (p = 0.01). From these numbers it can also be inferred that the relative proportion targeting deep layers (Va-VI) is slightly higher in RB (78.4%) than RS cells (69.2%). Consequently, RS cells target the superficial layers (I-IV) with 30.8% whereas only 21.6% of the RB cell axons are found there. Overall, both cell types form most of their output synapses in deeper layers (p<0.001). These differences are visualized in the histograms of Fig 6 and S3 Fig.

## Discussion

Concepts of cortical circuitry are constantly being refined [8,9,13,46,47]. The classical notion of a sequential canonical microcircuit (LIV projecting to LII/III that projects to LVa/b) in which layer Vb is considered the major final common output pathway of a cortical module [15,48] has recently been complemented by a set of data suggesting that layer Vb (at the same time) could serve as a “fast-in, fast-out” connection hub of a cortical column [49,50]. Our present results suggest that layer Vb-pyramidal cells of both types, RS and RB, should be involved in sensory-motor transformations that make use of the sequential pathway as well as the novel shortcut pathway [6],. At the input side, this is reflected by the large dendritic arbors of layer Vb pyramidal cells that perfectly suit associative signal integration by bottom-up sensory and top-down modulatory/attentional pathways [45]. This happens in a more column-restricted manner in RS cells whereas for RB cells it is typical that they integrate information from neighboring columns as well. At the output side, a similar distinction is visible. Within their home column, the axons of RS and RB prefer the deep layers but RS cells also strongly innervate the superficial layers. Within the deep layers, RB cells send a higher proportion of their axon into neighboring columns, driving synchronization of larger cell assemblies [51].

### Electrophysiology

Action potential firing properties of cells are important for delivering axonal outputs with different coding schemes. Therefore, we will discuss here the action potential firing patterns and the motivation for introducing a novel term. A multitude on terms for different or even the same patterns has been used in the past [36,52–55]. However, the two most reliable patterns with the most frequent occurrence were regular spiking (RS) and intrinsically burst spiking (IB). Since some groups did not encounter the IB pattern in their slice experiments (usually being parasagittal slices of young rats; [31,56]), the terms slender-tufted for assumed RS and thick-tufted for assumed IB cells is also frequently found in the literature, recently especially from in vivo studies (for reasons see below) [17,44,50]. Although there is a hypothesis that the “history” of the slice may dictate whether bursting cells can be found or not [57], in all our experiments, when aimed at pyramidal-shaped, large somata, we recorded bursting neurons without any special conditions being necessary. Since two other firing patterns consistently occurred that are characterized by an initial doublet (as the smallest form of a burst) followed by a series of single spikes, we wanted to coin an unambiguous term for “real bursters”, hence “repetitive bursting (RB)”. These respond to just suprathreshold and any stronger stimulation with a series of burst (usually in the form of doublets, rarely in triplets). Interestingly, a series of studies in the rat visual cortex [23,58] showed bursting neurons with an initial 3–5 spikes riding on a depolarizing envelope, followed by single spikes. Although they correlated this firing pattern with “thick-tufted” pyramidal cells, these neurons appear similar to our regular spiking ones in terms of “tuft complexity” (i.e. slender-tufted). On the other hand, their regular spiking “slender tufted” neurons are very similar to cortico-callosal neurons (which also project to the striatum and now are called intra-telencephalic neurons; see [13]) and lacked a terminal tuft of the apical dendrite [59]. This seems to be true for auditory cortex as well [25].

### Morphology

#### Somatodendritic domain

We consider soma shape and size to be a corollary of the size and configuration of the dendritic arbor, so that somatic properties do not need to be discussed here. The dendritic arbor, in turn, is considered to profoundly influence the action potential firing pattern [60]. In agreement with this modeling study we found a very high correlation between dendritic morphology and action potential firing pattern, however, the differences were of quantitative and not qualitative nature. RS layer Vb pyramidal cells possessed a thinner apical dendrite from which lesser oblique side branches went off and which ended in layer I with a narrower terminal tuft. RB layer Vb pyramidal cells, by contrast, had a much thicker apical dendrite with more oblique side branches and a very broad terminal tuft [21–25,31,53].

Furthermore, in both types, several basal dendrites can be found for each cell. Interestingly, we show that the oblique side branches of the apical dendrite, which are nearly exclusively originating close to the soma, add to the sphere of dendrites that is formed by the population of basal dendrites. Altogether, these branches form a very dense spherical dendritic arbor, which is mainly located in layer Vb and can be considered as one of two hotspots for inputs, the other one being found in layer I. What this means computationally, is at present unclear but it could be a cellular mechanism for associating bottom-up sensory with top-down contextual information [45].

Another consistent observation was that the horizontal extent of the dendritic tree was larger for RB than for RS cells, both, at the level of the soma (in layer Vb) and in layer I. Thus, RB cells could develop their large multi-whisker fields by transcolumnar connections but also by simply sending dendritic branches into the neighboring columns [50,61,62] where they potentially can be contacted by ascending thalamic axons [11,49]. This integrative capacity is also reflected by mesoscale cortical input maps [24] and their experience-dependent plasticity [39]. The increased plasticity might, at least partially, be enabled by the much lower level of inhibition that can functionally but not morphologically be detected for RB in comparison to RS pyramidal cells [24,35,37].

#### Axonal domain

A strong notion coming from in vitro studies is that RS cells have mainly or exclusively vertically oriented recurrent collaterals targeting layer I, whereas RB cells restrict their horizontal axonal collaterals to infragranular layers V/VI [22]. Here we showed that this qualitative difference does not hold when analyzing a population of recorded neurons selected for best morphological recovery of neurites. However, RS cells do preferentially target all layers of their home column whereas the overall much sparser local axonal arbor of RB cells has a stronger horizontal component. Most previous slice studies did neither attempt to fully reconstruct the axon, nor to relate its properties to the cortical layer and column organization. Thus it is difficult to directly compare our findings with published material, especially for RS cells. For RB cells, reports on thick-tufted layer V pyramidal cells basically agree with our finding that RB cells do target all supragranular layers [31,63].

A similar problem occurs when comparing our data to those of neurons recorded in vivo. Here the groups of Oberlaender and de Kock built an impressive quantitative framework and database for all excitatory neurons in rat barrel cortex [11,17,50,64]. However, due to their tangential cutting plane, precisely distinguishing layer Va from Vb is difficult. Thus, they “define” slender-tufted pyramidal neurons as being preferentially located in layer Va and thick-tufted neurons in layer Vb. Here we suggest that there exists a genuine population of layer Vb RS-pyramidal cells that is similar in dendritic morphology to their layer Va slender-tufted counterparts (see Fig 2 and also [39]) but different in axonal pattern. Whereas layer Va slender-tufted pyramidal cells strongly target supragranular layers in a broad column-overarching manner [64], layer Vb RS cells do so in a much more column-restricted manner (this study). In any case, it is interesting to note that the basic axonal pattern derived from our present slice study for RB cells is well in agreement with the in vivo data on thick-tufted neurons (L5tt; see Fig 3B in [17]). In addition, this study also revealed that layer V pyramidal cells have an interesting preference to project along arcs rather than rows [17] that was impossible for us to detect in the slice preparation.

## Conclusion

Layer Vb pyramidal cells have been studied extensively over the past two decades due to their crucial position in the cortical circuitry. However, a full quantitative description of physiologically-characterized neurons with respect to layers and columns was so far missing. Here we provide these data which firmly underscores the notion that RS and RB pyramidal cells do differ quantitatively but not qualitatively in terms of morphological properties. RS pyramidal cells have a stronger restriction of their input and output domains to their home column than RB cells, which extend a significant proportion of their processes into neighboring columns. This suggests that the different neuronal types are integrated into differential processing streams [13].

## Supporting Information

S1 Fig layer Vb firing patterns. **Classification of firing pattern types of layer Vb pyramidal cells.** Representative examples for two less-frequently observed firing pattern types of our sample. Voltage responses to rheobase stimulations (black) and just subthreshold (rheobase minus 1 pA) stimulations (gray) are shown in higher temporal resolution in the left panels. The entire voltage response to rheobase (black) and rheobase plus 50 pA (gray) stimulations are shown in the right panels. Voltage traces are always displayed above the corresponding rectangular current traces. **(A)** High-threshold bursting (HTB) neurons were similar to RS neurons at rheobase stimulations where they elicited one single AP. In response to clearly suprathreshold current pulses, however, they fired an initial burst consisting of two APs followed by an adapting train of single APs. **(B)** Initial doublet bursting (ID) neurons were indistinguishable from HTB neurons if depolarized with clearly suprathreshold current pulses: an initial burst was followed by an adapting train of single APs. However, ID neurons responded to rheobase stimulations with a doublet of APs in each case.
(JPG)

S2 Fig relative dendritic length. **Relative distribution of basal and apical dendrites across layers and columns in RS versus RB pyramidal cells (A)** Layer-independent summation of dendritic length with respect to home column or outside of it (extracolumnar). More than 90% of basal and apical dendrites are confined to the home column in both RS and RB (see color codes on the right hand side). Note, however, that there is more basal and apical dendrite in locations outside the home column for RB cells. **(B)** The relative distribution, independent of column borders, of the total length of basal dendrites and apical dendrite displays a similar laminar profile for RS and RB pyramidal cells, with obvious hot spots in layer Vb and I for apical dendrites and layer Vb for basal dendrites. Please note that layer Va is an additional site of preferential ramification of apical dendrites. **(C)** The distinction of dendrite distributed within (home column) and outside (extracolumnar) the home column shows a clear preference for the home column in both cell types. However, in layer Vb basal dendrites and in layer I apical dendrite of RB cells also extend substantially into the septum or even neighboring columns.
(JPG)

S3 Fig relative bouton numbers. **Relative distribution of axonal bouton number across layers and columns. (A)** Layer-independent summation of axonal bouton number with respect to home column or outside of it (extracolumnar) clearly shows that RS cell have a strong preference for their home column whereas RB cells display a nearly balanced distribution between home column and neighboring columns (see color codes on the right hand side) **(B)** The relative distribution of the total axonal bouton number, independent of column borders, presents a clear preference for infragranular layers Vb, VI and Va. **(C)** The distinction of boutons distributed within (home column) and outside (extracolumnar) the home column shows a preference for RB cells toward more extracolumnar boutons in all layers.
(JPG)

S1 Table Detailed somatodendritic properties. **Table A: Detailed somatodendritic properties of all reconstructed RS layer Vb-pyramidal cells.** All values are in μm (or μm^2^ for soma area) unless otherwise noted. Abbreviations: AD—apical dendrite; BD—basal dendrite; dend—dendrites; dia—diameter; hc—home column; hori—horizontal; max—maximum; min—minimum; nc—neighboring column; sept—septum. Roman numerals name cortical layers. **Table B: Detailed somatodendritic properties of all reconstructed RB layer Vb-pyramidal cells.** All values are in μm (or μm^2^ for soma area) unless otherwise noted. Abbreviations: AD—apical dendrite; BD—basal dendrite; dend—dendrites; dia—diameter; hc—home column; hori—horizontal; max—maximum; min—minimum; nc—neighboring column; sept—septum. Roman numerals name cortical layers.
(DOCX)

S2 Table Detailed axonal properties. **Table A: Detailed axonal properties of all reconstructed RS layer Vb-pyramidal cells.** All values are numbers (n) unless otherwise noted. Abbreviations: hc—home column; nc—neighboring column; sept—septum. Roman numerals name cortical layers. **Table B: Detailed axonal properties of all reconstructed RB layer Vb-pyramidal cells.** All values are numbers (n) unless otherwise noted. Abbreviations: hc—home column; nc—neighboring column; sept—septum. Roman numerals name cortical layers.
(DOCX)

S3 Table Mean_SD of values in Fig 3. Mean ± standard deviation of the values plotted in Fig 3.
(DOCX)

S4 Table Mean_SD of values in Fig 6. Mean ± standard deviation of the values plotted in Fig 6.
(DOCX)
