# Supplementary figures and images for: Morphological Characteristics of Electrophysiologically Characterized Layer Vb Pyramidal Cells in Rat Barrel Cortex
Source: PLoS One. 2016 Oct 5;11(10):e0164004. doi: 10.1371/journal.pone.0164004 (PMC5051735; doi:10.1371/journal.pone.0164004)

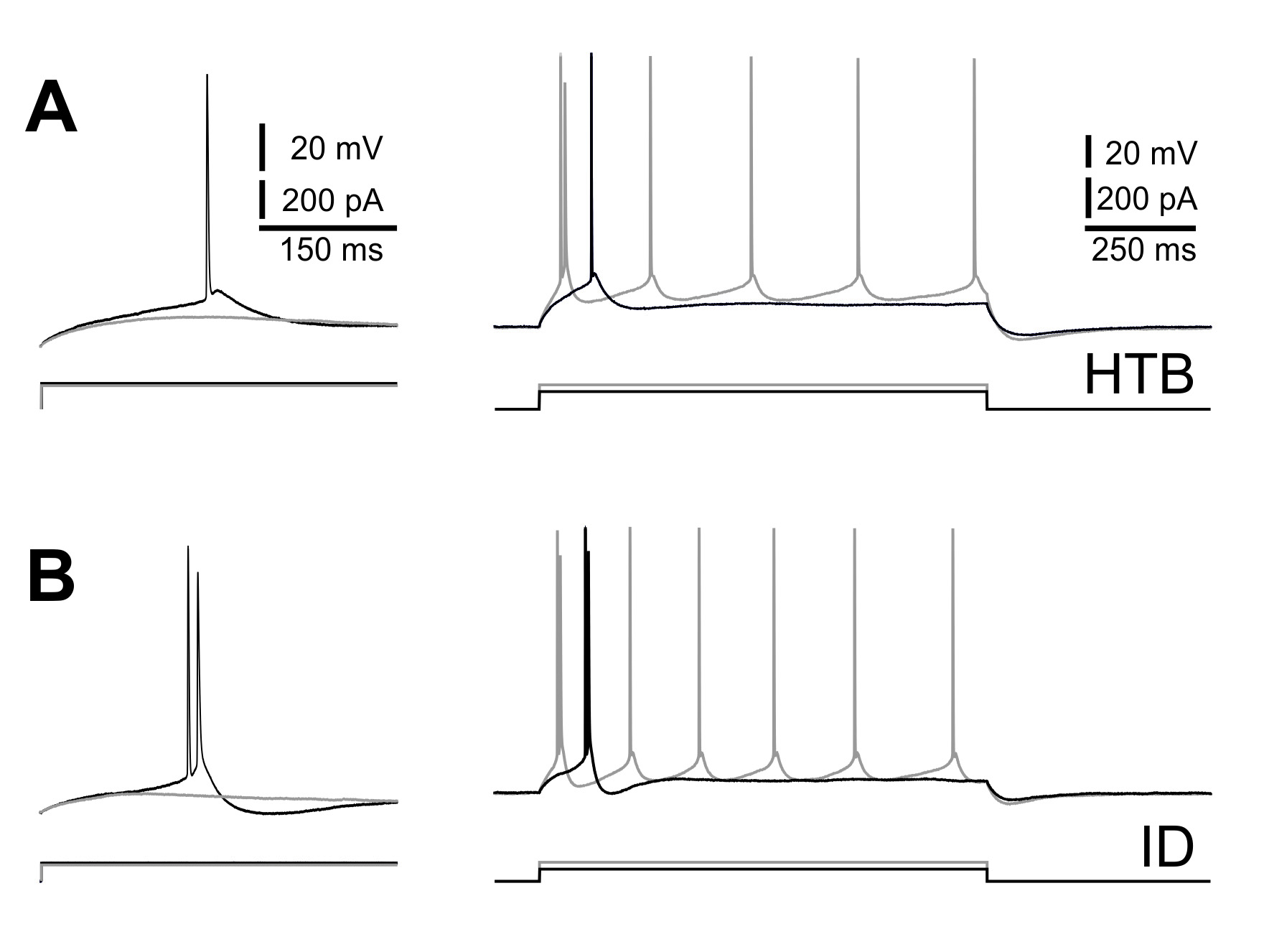

Supplement: S1 Fig — Classification of firing pattern types of layer Vb pyramidal cells. Representative examples for two less-frequently observed firing pattern types of our sample. Voltage responses to rheobase stimulations (black) and just subthreshold (rheobase minus 1 pA) stimulations (gray) are shown in higher temporal resolution in the left panels. The entire voltage response to rheobase (black) and rheobase plus 50 pA (gray) stimulations are shown in the right panels. Voltage traces are always displayed above the corresponding rectangular current traces. (A) High-threshold bursting (HTB) neurons were similar to RS neurons at rheobase stimulations where they elicited one single AP. In response to clearly suprathreshold current pulses, however, they fired an initial burst consisting of two APs followed by an adapting train of single APs. (B) Initial doublet bursting (ID) neurons were indistinguishable from HTB neurons if depolarized with clearly suprathreshold current pulses: an initial burst was followed by an adapting train of single APs. However, ID neurons responded to rheobase stimulations with a doublet of APs in each case. (JPG) [file pone.0164004.s001.jpg]

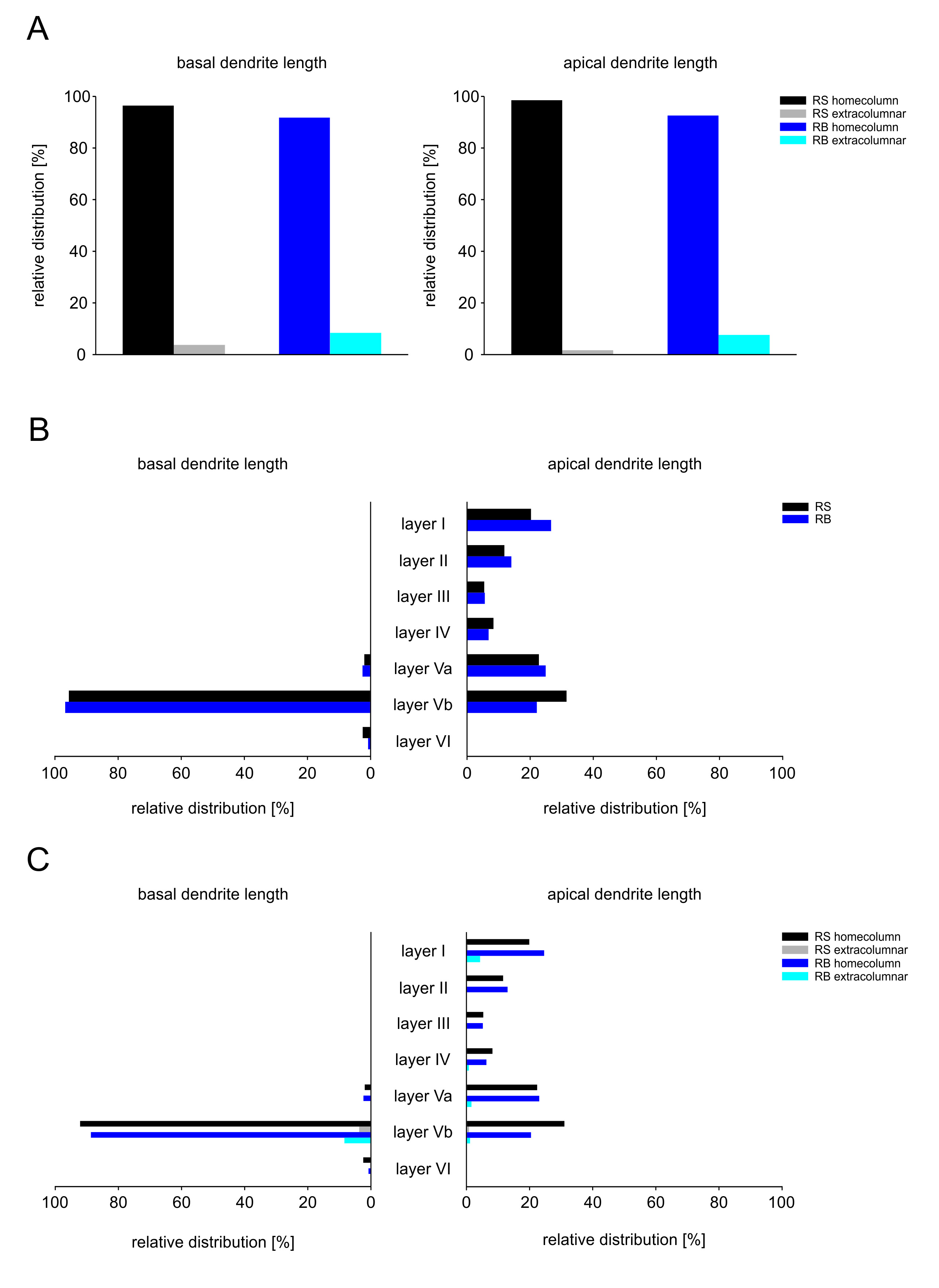

Supplement: S2 Fig — Relative distribution of basal and apical dendrites across layers and columns in RS versus RB pyramidal cells (A) Layer-independent summation of dendritic length with respect to home column or outside of it (extracolumnar). More than 90% of basal and apical dendrites are confined to the home column in both RS and RB (see color codes on the right hand side). Note, however, that there is more basal and apical dendrite in locations outside the home column for RB cells. (B) The relative distribution, independent of column borders, of the total length of basal dendrites and apical dendrite displays a similar laminar profile for RS and RB pyramidal cells, with obvious hot spots in layer Vb and I for apical dendrites and layer Vb for basal dendrites. Please note that layer Va is an additional site of preferential ramification of apical dendrites. (C) The distinction of dendrite distributed within (home column) and outside (extracolumnar) the home column shows a clear preference for the home column in both cell types. However, in layer Vb basal dendrites and in layer I apical dendrite of RB cells also extend substantially into the septum or even neighboring columns. (JPG) [file pone.0164004.s002.jpg]

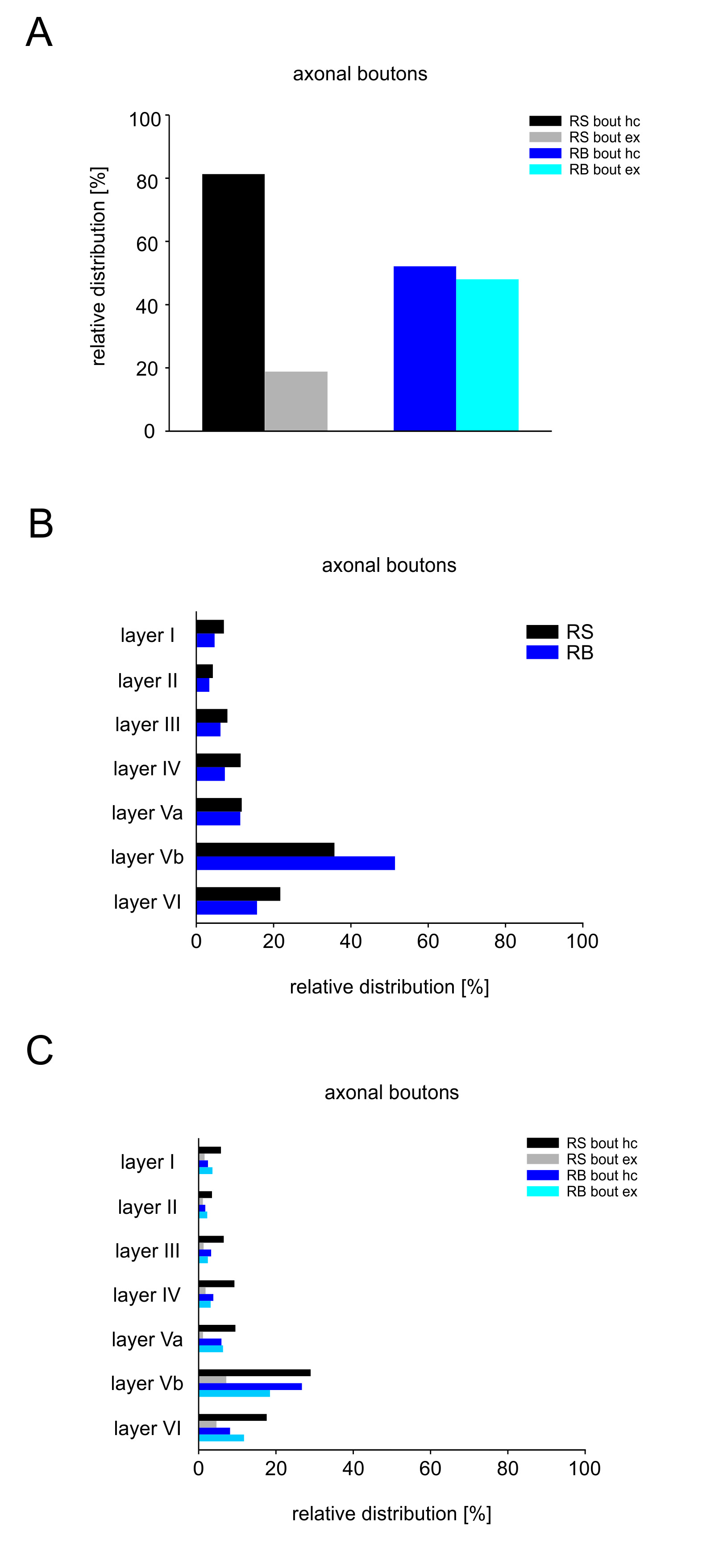

Supplement: S3 Fig — Relative distribution of axonal bouton number across layers and columns. (A) Layer-independent summation of axonal bouton number with respect to home column or outside of it (extracolumnar) clearly shows that RS cell have a strong preference for their home column whereas RB cells display a nearly balanced distribution between home column and neighboring columns (see color codes on the right hand side) (B) The relative distribution of the total axonal bouton number, independent of column borders, presents a clear preference for infragranular layers Vb, VI and Va. (C) The distinction of boutons distributed within (home column) and outside (extracolumnar) the home column shows a preference for RB cells toward more extracolumnar boutons in all layers. (JPG) [file pone.0164004.s003.jpg]
