# Supplementary material for: Morphological Characteristics of Electrophysiologically Characterized Layer Vb Pyramidal Cells in Rat Barrel Cortex
Source: PLoS One. 2016 Oct 5;11(10):e0164004. doi: 10.1371/journal.pone.0164004 (PMC5051735; doi:10.1371/journal.pone.0164004)
Supplement: S1 Table — Table A: Detailed somatodendritic properties of all reconstructed RS layer Vb-pyramidal cells. All values are in μm (or μm2 for soma area) unless otherwise noted. Abbreviations: AD—apical dendrite; BD—basal dendrite; dend—dendrites; dia—diameter; hc—home column; hori—horizontal; max—maximum; min—minimum; nc—neighboring column; sept—septum. Roman numerals name cortical layers. Table B: Detailed somatodendritic properties of all reconstructed RB layer Vb-pyramidal cells. All values are in μm (or μm2 for soma area) unless otherwise noted. Abbreviations: AD—apical dendrite; BD—basal dendrite; dend—dendrites; dia—diameter; hc—home column; hori—horizontal; max—maximum; min—minimum; nc—neighboring column; sept—septum. Roman numerals name cortical layers. (DOCX) [file pone.0164004.s004.docx]

| **Cell #** | **1** | **2** | **3** | **4** | **5** | **6** | **7** | **8** | **9** | **10** | **11** | **12** | **13** | **14** | **15** | **16** |
| --- | --- | --- | --- | --- | --- | --- | --- | --- | --- | --- | --- | --- | --- | --- | --- | --- |
| **Feret max** | 23 | 24 | 30 | 19 | 31 | 26 | 29 | 24 | 27 | 20 | 22 | 22 | 26 | 24 | 27 | 25 |
| **Feret min** | 14 | 16 | 18 | 14 | 20 | 17 | 22 | 17 | 21 | 12 | 15 | 18 | 20 | 17 | 15 | 20 |
| **Soma area** | 375 | 300 | 382 | 194 | 445 | 296 | 417 | 298 | 389 | 157 | 252 | 268 | 372 | 308 | 287 | 364 |
| **Dend span-vertical** | 1335 | 991 | 1212 | 1157 | 1042 | 967 | 1021 | 1032 | 1036 | 1406 | 1160 | 1086 | 1212 | 1277 | 1018 | 1312 |
| **Dend span-hori (AD tuft)** | 151 | 250 | 186 | 229 | 258 | 76 | 288 | 265 | 281 | 264 | 210 | 265 | 316 | 259 | 333 | 343 |
| **Dend span-hori (AD soma)** | 222 | 181 | 274 | 194 | 212 | 229 | 234 | 264 | 186 | 225 | 215 | 269 | 263 | 258 | 209 | 247 |
| **Dend span-hori (BD)** | 405 | 249 | 353 | 167 | 243 | 273 | 278 | 283 | 197 | 254 | 236 | 245 | 292 | 291 | 221 | 300 |
| **Primary dendrites (n)** | 5 | 5 | 5 | 7 | 8 | 7 | 4 | 5 | 7 | 7 | 5 | 4 | 9 | 6 | 8 | 7 |
| **BD-max dia** | 4.3 | 2.9 | 3.6 | 1.6 | 2.3 | 2.6 | 3.7 | 2.8 | 3.1 | 3.1 | 3.6 | 2.8 | 3.3 | 3.1 | 2.1 | 3.6 |
| **BD-min dia** | 3.2 | 1.5 | 1.8 | 1.1 | 1.8 | 2.1 | 3.0 | 1.4 | 1.0 | 1.1 | 2.0 | 2.3 | 1.2 | 1.7 | 1.5 | 1.7 |
| **AD-max dia** | 3.6 | 5.0 | 4.5 | 4.1 | 5.6 | 3.8 | 4.3 | 4.6 | 4.7 | 3.6 | 4.9 | 4.5 | 4.5 | 5.1 | 4.5 | 5.9 |
| **BD-ends** | 25 | 23 | 20 | 21 | 32 | 23 | 31 | 22 | 27 | 19 | 22 | 23 | 36 | 27 | 15 | 27 |
| **AD-ends** | 23 | 45 | 30 | 45 | 46 | 23 | 44 | 41 | 49 | 40 | 39 | 24 | 57 | 46 | 45 | 53 |
| **Total-ends** | 48 | 68 | 50 | 66 | 78 | 46 | 75 | 63 | 76 | 59 | 61 | 47 | 93 | 73 | 60 | 80 |
| **BD-length** | 3834 | 2345 | 3273 | 1594 | 2843 | 2417 | 3992 | 3788 | 2484 | 2726 | 2782 | 3090 | 4565 | 3808 | 1980 | 3445 |
| **AD-length** | 4432 | 6470 | 5276 | 6478 | 6756 | 3776 | 6798 | 8391 | 8194 | 6192 | 6363 | 4475 | 9700 | 7211 | 7248 | 9542 |
| **Total-dendritic length** | 8266 | 8815 | 8549 | 8072 | 9599 | 6193 | 10790 | 12179 | 10678 | 8918 | 9145 | 7565 | 14265 | 11019 | 9228 | 12987 |
| **BD-Va hc** | 115 | 0 | 0 | 0 | 0 | 364 | 367 | 79 | 0 | 0 | 0 | 0 | 0 | 0 | 0 | 0 |
| **BD-Vb hc** | 3555 | 1870 | 3080 | 1139 | 2793 | 2027 | 3622 | 3507 | 2484 | 2483 | 2782 | 3090 | 4528 | 2465 | 1980 | 3445 |
| **BD-VI hc** | 0 | 36 | 89 | 0 | 50 | 0 | 0 | 130 | 0 | 0 | 0 | 0 | 35 | 818 | 0 | 0 |
| **BD-Vb sept** | 170 | 251 | 93 | 203 | 0 | 31 | 0 | 65 | 0 | 92 | 0 | 0 | 0 | 364 | 0 | 0 |
| **BD-Vb nc** | 0 | 167 | 0 | 43 | 0 | 0 | 0 | 0 | 0 | 140 | 0 | 0 | 0 | 161 | 0 | 0 |
| **AD-I hc** | 885 | 1175 | 1557 | 1301 | 1668 | 309 | 1826 | 1166 | 1043 | 1253 | 502 | 890 | 2005 | 644 | 2248 | 2767 |
| **AD-I sept** | 0 | 254 | 0 | 0 | 0 | 0 | 0 | 0 | 0 | 0 | 0 | 0 | 0 | 0 | 24 | 43 |
| **AD-I nc** | 0 | 149 | 0 | 0 | 0 | 0 | 0 | 0 | 0 | 0 | 0 | 0 | 0 | 0 | 0 |  |
| **AD-II hc** | 417 | 1147 | 304 | 984 | 717 | 370 | 312 | 741 | 1704 | 906 | 785 | 257 | 1065 | 777 | 713 | 1185 |
| **AD-III hc** | 262 | 248 | 211 | 334 | 209 | 243 | 257 | 364 | 576 | 376 | 493 | 244 | 564 | 244 | 398 | 672 |
| **AD-IV hc** | 486 | 291 | 372 | 522 | 531 | 633 | 1158 | 811 | 820 |  | 449 | 319 | 743 | 570 | 365 | 703 |
| **AD-IV sept** | 0 | 0 | 0 | 0 | 0 | 0 | 0 | 56 | 0 | 0 | 0 | 0 | 0 | 0 | 0 | 0 |
| **AD-IV nc** | 0 | 0 | 0 | 0 | 0 | 0 | 0 | 29 | 0 | 0 | 0 | 0 | 0 | 0 | 0 | 0 |
| **AD-Va hc** | 776 | 1213 | 792 | 952 | 1030 | 2224 | 2833 | 3238 | 938 | 500 | 1686 | 1355 | 1994 | 1257 | 1330 | 1750 |
| **AD-Va sept** | 0 | 73 | 0 | 4 | 0 | 0 | 0 | 185 | 0 | 0 | 0 | 0 | 0 | 0 | 0 | 0 |
| **AD-Va nc** | 0 | 0 | 0 | 0 | 0 | 0 | 0 | 0 | 0 | 0 | 0 | 0 | 0 | 0 | 0 | 0 |
| **AD-Vb hc** | 1604 | 1627 | 2037 | 1979 | 2584 | 0 | 412 | 1777 | 3080 | 2651 | 2447 | 1403 | 3325 | 3531 | 2192 | 2421 |
| **AD-Vb sept** | 0 | 105 | 0 | 268 | 0 | 0 | 0 | 12 | 0 | 75 | 0 | 0 | 0 | 175 | 0 | 0 |
| **AD-Vb nc** | 0 | 0 | 0 | 0 | 0 | 0 | 0 | 0 | 0 | 164 | 0 | 0 | 0 | 70 | 0 | 0 |

**Supplementary Table 1a: Detailed somatodendritic properties of all reconstructed RS layer Vb-pyramidal cells.**

All values are in µm (or µm^2^ for soma area) unless otherwise noted. Abbreviations: AD – apical dendrite; BD – basal dendrite; dend – dendrites; dia – diameter; hc – home column; hori – horizontal; max – maximum; min – minimum; nc – neighboring column; sept – septum. Roman numerals name cortical layers.

| **Cell #** | **1** | **2** | **3** | **4** | **5** | **6** | **7** | **8** | **9** | **10** | **11** | **12** | **13** | **14** | **15** | **16** | **17** |
| --- | --- | --- | --- | --- | --- | --- | --- | --- | --- | --- | --- | --- | --- | --- | --- | --- | --- |
| **Feret max** | 37 | 33 | 28 | 32 | 35 | 31 | 36 | 28 | 31 | 31 | 25 | 30 | 33 | 34 | 31 | 27 | 31 |
| **Feret min** | 24 | 21 | 20 | 17 | 21 | 19 | 20 | 20 | 21 | 21 | 18 | 17 | 25 | 20 | 19 | 19 | 17 |
| **Soma area** | 644 | 485 | 388 | 403 | 513 | 383 | 474 | 362 | 477 | 468 | 340 | 368 | 522 | 464 | 314 | 339 | 370 |
| **Dend span-vertical** | 1000 | 996 | 1150 | 1146 | 1289 | 1264 | 1119 | 936 | 1138 | 1126 | 1126 | 1122 | 1176 | 1154 | 1030 | 1146 | 1164 |
| **Dend span-hori (AD tuft)** | 527 | 497 | 553 | 253 | 419 | 403 | 369 | 297 | 398 | 559 | 403 | 494 | 598 | 404 | 492 | 514 | 430 |
| **Dend span-hori (AD soma)** | 331 | 262 | 271 | 268 | 311 | 261 | 229 | 199 | 334 | 271 | 393 | 302 | 353 | 332 | 341 | 299 | 315 |
| **Dend span-hori (BD)** | 365 | 326 | 385 | 322 | 485 | 367 | 340 | 273 | 428 | 369 | 431 | 302 | 399 | 402 | 391 | 335 | 358 |
| **Primary dendrites (n)** | 7 | 5 | 7 | 7 | 8 | 5 | 7 | 7 | 6 | 6 | 6 | 7 | 6 | 6 | 6 | 7 | 7 |
| **BD-max dia** | 4.2 | 4.3 | 3.4 | 2.8 | 2.9 | 3.9 | 3.8 | 2.9 | 3.9 | 5.1 | 3.8 | 4.1 | 4.6 | 4.5 | 4.5 | 4.2 | 5.4 |
| **BD-min dia** | 2.8 | 2.7 | 2.2 | 1.5 | 2.3 | 2.5 | 1.6 | 2.1 | 2.7 | 2.7 | 2.2 | 2.6 | 3.8 | 3.6 | 2.1 | 2.0 | 3.2 |
| **AD-max dia** | 6.0 | 6.9 | 7.6 | 5.7 | 6.6 | 5.4 | 6.8 | 4.9 | 7.9 | 6.6 | 5.4 | 7.3 | 6.4 | 6.4 | 4.6 | 7.0 | 7.8 |
| **BD-ends** | 46 | 24 | 40 | 30 | 45 | 29 | 32 | 30 | 39 | 51 | 34 | 33 | 42 | 36 | 36 | 36 | 50 |
| **AD-ends** | 54 | 64 | 42 | 42 | 53 | 54 | 43 | 39 | 58 | 48 | 43 | 46 | 62 | 35 | 44 | 59 | 40 |
| **Total-ends** | 100 | 88 | 88 | 72 | 98 | 83 | 75 | 69 | 97 | 99 | 77 | 79 | 104 | 71 | 80 | 95 | 90 |
| **BD-length** | 5605 | 3304 | 6215 | 3918 | 6169 | 3775 | 5545 | 3778 | 6251 | 8589 | 4694 | 5396 | 5997 | 6280 | 5151 | 4719 | 7772 |
| **AD-length** | 9012 | 8975 | 7703 | 7340 | 8177 | 10625 | 8342 | 6990 | 10120 | 7754 | 8213 | 9143 | 12022 | 6864 | 7750 | 9609 | 7500 |
| **Total-dendritic length** | 14617 | 12279 | 13918 | 11258 | 14346 | 14400 | 13887 | 10768 | 16371 | 16343 | 12907 | 14539 | 18019 | 13144 | 12901 | 14328 | 15272 |
| **BD-Va hc** | 0 | 0 | 0 | 0 | 0 | 11 | 127 | 0 | 733 | 138 | 36 | 0 | 358 | 24 | 0 | 10 | 707 |
| **BD-Vb hc** | 5164 | 3032 | 5977 | 3733 | 5383 | 3764 | 5255 | 3429 | 3373 | 8349 | 4269 | 5396 | 5541 | 5029 | 5048 | 4481 | 5192 |
| **BD-VI hc** | 81 | 78 | 64 | 0 | 74 | 0 | 0 | 0 | 0 | 0 | 34 | 0 | 0 | 301 | 0 | 12 | 0 |
| **BD-Vb sept** | 304 | 197 | 111 | 180 | 536 | 0 | 142 | 145 | 1537 | 102 | 315 | 0 | 30 | 548 | 89 | 180 | 948 |
| **BD-Vb nc** | 52 | 0 | 50 | 0 | 175 | 0 | 21 | 175 | 543 | 0 | 29 | 0 | 0 | 363 | 14 | 41 | 927 |
| **AD-I hc** | 3073 | 1460 | 2653 | 1984 | 1249 | 2116 | 2215 | 537 | 2087 | 2014 | 1561 | 1882 | 4467 | 1434 | 2657 | 2597 | 1825 |
| **AD-I sept** | 75 | 48 | 147 | 0 | 70 | 0 | 0 | 12 | 983 | 115 | 10 | 240 | 654 | 492 | 188 | 359 | 558 |
| **AD-I nc** | 24 | 30 | 250 | 0 | 0 | 0 | 0 | 0 | 184 | 0 | 0 | 25 | 82 | 180 | 463 | 388 | 447 |
| **AD-II hc** | 571 | 2465 | 989 | 1011 | 1603 | 2137 | 682 | 1578 | 562 | 613 | 2782 | 1115 | 423 | 278 | 679 | 681 | 761 |
| **AD-III hc** | 327 | 573 | 464 | 498 | 786 | 577 | 279 | 881 | 239 | 235 | 523 | 671 | 225 | 466 | 214 | 250 | 312 |
| **AD-IV hc** | 550 | 369 | 280 | 528 | 406 | 1055 | 341 | 514 | 737 | 915 | 671 | 480 | 833 | 290 | 468 | 271 | 428 |
| **AD-IV sept** | 0 | 0 | 0 | 0 | 0 | 0 | 0 | 0 | 950 | 0 | 0 | 0 | 0 | 0 | 0 | 0 | 0 |
| **AD-IV nc** | 0 | 0 | 0 | 0 | 0 | 0 | 0 | 0 | 125 | 0 | 0 | 0 | 0 | 0 | 0 | 0 | 0 |
| **AD-Va hc** | 1276 | 950 | 1790 | 1140 | 2484 | 2195 | 4041 | 960 | 2209 | 2788 | 934 | 1605 | 3382 | 2106 | 1887 | 1658 | 2099 |
| **AD-Va sept** | 0 | 0 | 0 | 0 | 0 | 0 | 7 | 104 | 1214 | 0 | 19 | 0 | 0 | 142 | 41 | 16 | 329 |
| **AD-Va nc** | 0 | 0 | 0 | 0 | 0 | 0 | 0 | 83 | 53 | 0 | 0 | 0 | 0 | 0 | 26 | 0 | 211 |
| **AD-Vb hc** | 2962 | 2457 | 1052 | 1763 | 1503 | 2664 | 764 | 2092 | 201 | 1974 | 1487 | 3122 | 1961 | 1323 | 1096 | 2827 | 478 |
| **AD-Vb sept** | 104 | 192 | 9 | 84 | 76 | 0 | 0 | 30 | 81 | 0 | 229 | 0 | 0 | 122 | 18 | 317 | 0 |
| **AD-Vb nc** | 0 | 0 | 0 | 0 | 0 | 0 | 0 | 0 | 0 | 0 | 0 | 0 | 0 | 3 | 0 | 250 | 50 |

**Supplementary Table 1b: Detailed somatodendritic properties of all reconstructed RB layer Vb-pyramidal cells.**

All values are in µm (or µm^2^ for soma area) unless otherwise noted. Abbreviations: AD – apical dendrite; BD – basal dendrite; dend – dendrites; dia – diameter; hc – home column; hori – horizontal; max – maximum; min – minimum; nc – neighboring column; sept – septum. Roman numerals name cortical layers.

| **Cell #** | **1** | **2** | **3** | **4** | **5** | **6** | **7** | **8** | **9** | **10** | **11** | **12** |
| --- | --- | --- | --- | --- | --- | --- | --- | --- | --- | --- | --- | --- |
| **Horizontal spread (µm)** | 2309 | 575 | 809 | 1356 | 489 | 831 | 934 | 953 | 829 | 1113 | 766 | 1255 |
| **Total length (µm)** | 10870 | 9677 | 19164 | 10059 | 18515 | 15414 | 16894 | 8686 | 18888 | 23428 | 14576 | 24674 |
| **Nodes (n)** | 48 | 50 | 99 | 37 | 79 | 73 | 49 | 46 | 91 | 98 | 44 | 100 |
| **Boutons (n)** | 1960 | 1640 | 5239 | 2239 | 4067 | 5065 | 3467 | 1767 | 4720 | 5582 | 2572 | 5410 |
| **Bouton density** | 18.0 | 16.9 | 27.3 | 22.3 | 22.0 | 32.9 | 20.5 | 20.3 | 25.0 | 23.8 | 17.6 | 21.9 |
| **I hc** | 0 | 166 | 167 | 0 | 1338 | 28 | 83 | 18 | 147 | 81 | 88 | 399 |
| **I sept** | 31 | 9 | 15 | 0 | 106 | 0 | 0 | 0 | 0 | 25 | 0 | 53 |
| **I nc** | 288 | 0 | 0 | 0 | 63 | 0 | 0 | 0 | 0 | 0 | 0 | 91 |
| **II hc** | 32 | 64 | 126 | 86 | 373 | 138 | 131 | 90 | 186 | 136 | 65 | 84 |
| **II sept** | 24 | 0 | 0 | 32 | 0 | 0 | 0 | 0 | 0 | 68 | 0 | 0 |
| **II nc** | 108 | 0 | 32 | 130 | 0 | 0 | 0 | 75 | 0 | 11 | 0 | 13 |
| **III hc** | 81 | 47 | 241 | 254 | 395 | 371 | 421 | 60 | 352 | 188 | 99 | 332 |
| **III sept** | 13 | 0 | 13 | 15 | 0 | 0 | 0 | 57 | 72 | 68 | 0 | 33 |
| **III nc** | 2 | 0 | 31 | 7 | 44 | 0 | 0 | 28 | 11 | 56 | 79 | 36 |
| **IV hc** | 46 | 100 | 450 | 193 | 360 | 831 | 589 | 173 | 361 | 359 | 196 | 386 |
| **IV sept** | 0 | 15 | 34 | 0 | 8 | 0 | 0 | 36 | 131 | 156 | 0 | 36 |
| **IV nc** | 0 | 0 | 0 | 0 | 38 | 0 | 0 | 0 | 62 | 137 | 84 | 50 |
| **Va hc** | 37 | 109 | 564 | 359 | 563 | 396 | 463 | 378 | 391 | 466 | 97 | 326 |
| **Va sept** | 0 | 16 | 0 | 23 | 46 | 0 | 0 | 0 | 19 | 55 | 0 | 9 |
| **Va nc** | 24 | 18 | 0 | 38 | 56 | 0 | 0 | 0 | 63 | 0 | 86 | 45 |
| **Vb hc** | 86 | 534 | 1966 | 461 | 505 | 2308 | 985 | 725 | 1324 | 1434 | 573 | 1731 |
| **Vb sept** | 63 | 35 | 114 | 18 | 27 | 331 | 21 | 0 | 152 | 120 | 65 | 313 |
| **Vb nc** | 321 | 0 | 7 | 261 | 13 | 55 | 55 | 0 | 229 | 76 | 225 | 627 |
| **VI hc** | 320 | 457 | 1313 | 142 | 94 | 596 | 437 | 727 | 1123 | 1504 | 609 | 355 |
| **VI sept** | 81 | 10 | 80 | 0 | 15 | 0 | 23 | 0 | 16 | 174 | 70 | 59 |
| **VI nc** | 182 | 0 | 51 | 38 | 12 | 0 | 189 | 0 | 54 | 431 | 188 | 347 |

**Supplementary Table 2a: Detailed axonal properties of all reconstructed RS layer Vb-pyramidal cells.**

All values are numbers (n) unless otherwise noted. Abbreviations: hc – home column; nc – neighboring column; sept – septum. Roman numerals name cortical layers.

| **Cell #** | **1** | **2** | **3** | **4** | **5** | **6** | **7** | **8** | **9** | **10** | **11** | **12** | **13** |
| --- | --- | --- | --- | --- | --- | --- | --- | --- | --- | --- | --- | --- | --- |
| **Horizontal spread** | 888 | 1625 | 1333 | 1060 | 1497 | 1828 | 1146 | 1613 | 936 | 1461 | 1854 | 1122 | 1754 |
| **Total length** | 8115 | 11185 | 4918 | 6687 | 8409 | 16672 | 12019 | 13799 | 8254 | 5305 | 11985 | 5812 | 11596 |
| **Nodes (n)** | 28 | 46 | 14 | 18 | 42 | 91 | 58 | 58 | 29 | 18 | 45 | 22 | 48 |
| **Boutons (n)** | 1758 | 2293 | 934 | 1344 | 1500 | 3526 | 2619 | 3084 | 1441 | 1171 | 2434 | 1023 | 2657 |
| **Bouton density** | 21.7 | 20.5 | 19.0 | 20.1 | 17.8 | 21.1 | 21.8 | 22.3 | 17.5 | 22.1 | 20.3 | 17.6 | 22.9 |
| **I hc** | 65 | 12 | 106 | 0 | 0 | 21 | 0 | 133 | 9 | 0 | 62 | 29 | 138 |
| **I sept** | 0 | 0 | 0 | 10 | 0 | 65 | 184 | 14 | 42 | 0 | 0 | 0 | 0 |
| **I nc** | 0 | 0 | 0 | 56 | 0 | 145 | 133 | 159 | 0 | 45 | 0 | 0 | 0 |
| **II hc** | 82 | 14 | 37 | 0 | 0 | 43 | 28 | 44 | 0 | 0 | 66 | 44 | 53 |
| **II sept** | 0 | 0 | 0 | 0 | 0 | 32 | 99 | 30 | 39 | 0 | 0 | 0 | 0 |
| **II nc** | 0 | 0 | 0 | 113 | 0 | 48 | 34 | 94 | 0 | 45 | 0 | 0 | 0 |
| **III hc** | 72 | 29 | 117 | 0 | 0 | 86 | 145 | 102 | 0 | 0 | 72 | 98 | 47 |
| **III sept** | 0 | 0 | 0 | 0 | 0 | 4 | 43 | 4 | 37 | 0 | 0 | 0 | 0 |
| **III nc** | 0 | 0 | 0 | 137 | 0 | 0 | 0 | 298 | 0 | 44 | 0 | 0 | 0 |
| **IV hc** | 66 | 71 | 62 | 0 | 0 | 220 | 129 | 109 | 0 | 0 | 111 | 58 | 81 |
| **IV sept** | 0 | 0 | 0 | 0 | 45 | 0 | 30 | 49 | 73 | 0 | 0 | 0 | 0 |
| **IV nc** | 36 | 0 | 0 | 166 | 11 | 0 | 0 | 222 | 35 | 70 | 0 | 0 | 0 |
| **Va hc** | 89 | 119 | 36 | 57 | 0 | 243 | 97 | 144 | 126 | 0 | 316 | 65 | 109 |
| **Va sept** | 9 | 56 | 17 | 12 | 20 | 0 | 4 | 47 | 66 | 0 | 28 | 51 | 42 |
| **Va nc** | 163 | 91 | 69 | 129 | 100 | 4 | 0 | 203 | 47 | 30 | 97 | 119 | 94 |
| **Vb hc** | 501 | 435 | 233 | 325 | 244 | 934 | 814 | 281 | 223 | 274 | 765 | 321 | 984 |
| **Vb sept** | 62 | 102 | 64 | 32 | 147 | 84 | 95 | 48 | 63 | 27 | 205 | 37 | 165 |
| **Vb nc** | 391 | 449 | 86 | 140 | 451 | 397 | 217 | 175 | 233 | 181 | 213 | 51 | 263 |
| **VI hc** | 8 | 275 | 6 | 147 | 77 | 141 | 294 | 262 | 133 | 106 | 163 | 32 | 289 |
| **VI sept** | 9 | 90 | 0 | 11 | 0 | 255 | 61 | 64 | 14 | 17 | 26 | 0 | 89 |
| **VI nc** | 204 | 422 | 0 | 0 | 0 | 629 | 108 | 280 | 52 | 130 | 77 | 0 | 243 |

**Supplementary Table 2b: Detailed axonal properties of all reconstructed RB layer Vb-pyramidal cells.**

All values are numbers (n) unless otherwise noted. Abbreviations: hc – home column; nc – neighboring column; sept – septum. Roman numerals name cortical layers.
