# Supplementary material for: Morphological Characteristics of Electrophysiologically Characterized Layer Vb Pyramidal Cells in Rat Barrel Cortex
Source: PLoS One. 2016 Oct 5;11(10):e0164004. doi: 10.1371/journal.pone.0164004 (PMC5051735; doi:10.1371/journal.pone.0164004)
Supplement: S2 Table — Table A: Detailed axonal properties of all reconstructed RS layer Vb-pyramidal cells. All values are numbers (n) unless otherwise noted. Abbreviations: hc—home column; nc—neighboring column; sept—septum. Roman numerals name cortical layers. Table B: Detailed axonal properties of all reconstructed RB layer Vb-pyramidal cells. All values are numbers (n) unless otherwise noted. Abbreviations: hc—home column; nc—neighboring column; sept—septum. Roman numerals name cortical layers. (DOCX) [file pone.0164004.s005.docx]

| **Cell #** | **1** | **2** | **3** | **4** | **5** | **6** | **7** | **8** | **9** | **10** | **11** | **12** |
| --- | --- | --- | --- | --- | --- | --- | --- | --- | --- | --- | --- | --- |
| **Horizontal spread (µm)** | 2309 | 575 | 809 | 1356 | 489 | 831 | 934 | 953 | 829 | 1113 | 766 | 1255 |
| **Total length (µm)** | 10870 | 9677 | 19164 | 10059 | 18515 | 15414 | 16894 | 8686 | 18888 | 23428 | 14576 | 24674 |
| **Nodes (n)** | 48 | 50 | 99 | 37 | 79 | 73 | 49 | 46 | 91 | 98 | 44 | 100 |
| **Boutons (n)** | 1960 | 1640 | 5239 | 2239 | 4067 | 5065 | 3467 | 1767 | 4720 | 5582 | 2572 | 5410 |
| **Bouton density** | 18.0 | 16.9 | 27.3 | 22.3 | 22.0 | 32.9 | 20.5 | 20.3 | 25.0 | 23.8 | 17.6 | 21.9 |
| **I hc** | 0 | 166 | 167 | 0 | 1338 | 28 | 83 | 18 | 147 | 81 | 88 | 399 |
| **I sept** | 31 | 9 | 15 | 0 | 106 | 0 | 0 | 0 | 0 | 25 | 0 | 53 |
| **I nc** | 288 | 0 | 0 | 0 | 63 | 0 | 0 | 0 | 0 | 0 | 0 | 91 |
| **II hc** | 32 | 64 | 126 | 86 | 373 | 138 | 131 | 90 | 186 | 136 | 65 | 84 |
| **II sept** | 24 | 0 | 0 | 32 | 0 | 0 | 0 | 0 | 0 | 68 | 0 | 0 |
| **II nc** | 108 | 0 | 32 | 130 | 0 | 0 | 0 | 75 | 0 | 11 | 0 | 13 |
| **III hc** | 81 | 47 | 241 | 254 | 395 | 371 | 421 | 60 | 352 | 188 | 99 | 332 |
| **III sept** | 13 | 0 | 13 | 15 | 0 | 0 | 0 | 57 | 72 | 68 | 0 | 33 |
| **III nc** | 2 | 0 | 31 | 7 | 44 | 0 | 0 | 28 | 11 | 56 | 79 | 36 |
| **IV hc** | 46 | 100 | 450 | 193 | 360 | 831 | 589 | 173 | 361 | 359 | 196 | 386 |
| **IV sept** | 0 | 15 | 34 | 0 | 8 | 0 | 0 | 36 | 131 | 156 | 0 | 36 |
| **IV nc** | 0 | 0 | 0 | 0 | 38 | 0 | 0 | 0 | 62 | 137 | 84 | 50 |
| **Va hc** | 37 | 109 | 564 | 359 | 563 | 396 | 463 | 378 | 391 | 466 | 97 | 326 |
| **Va sept** | 0 | 16 | 0 | 23 | 46 | 0 | 0 | 0 | 19 | 55 | 0 | 9 |
| **Va nc** | 24 | 18 | 0 | 38 | 56 | 0 | 0 | 0 | 63 | 0 | 86 | 45 |
| **Vb hc** | 86 | 534 | 1966 | 461 | 505 | 2308 | 985 | 725 | 1324 | 1434 | 573 | 1731 |
| **Vb sept** | 63 | 35 | 114 | 18 | 27 | 331 | 21 | 0 | 152 | 120 | 65 | 313 |
| **Vb nc** | 321 | 0 | 7 | 261 | 13 | 55 | 55 | 0 | 229 | 76 | 225 | 627 |
| **VI hc** | 320 | 457 | 1313 | 142 | 94 | 596 | 437 | 727 | 1123 | 1504 | 609 | 355 |
| **VI sept** | 81 | 10 | 80 | 0 | 15 | 0 | 23 | 0 | 16 | 174 | 70 | 59 |
| **VI nc** | 182 | 0 | 51 | 38 | 12 | 0 | 189 | 0 | 54 | 431 | 188 | 347 |

**Supplementary Table 2a: Detailed axonal properties of all reconstructed RS layer Vb-pyramidal cells.**

All values are numbers (n) unless otherwise noted. Abbreviations: hc – home column; nc – neighboring column; sept – septum. Roman numerals name cortical layers.

| **Cell #** | **1** | **2** | **3** | **4** | **5** | **6** | **7** | **8** | **9** | **10** | **11** | **12** | **13** |
| --- | --- | --- | --- | --- | --- | --- | --- | --- | --- | --- | --- | --- | --- |
| **Horizontal spread** | 888 | 1625 | 1333 | 1060 | 1497 | 1828 | 1146 | 1613 | 936 | 1461 | 1854 | 1122 | 1754 |
| **Total length** | 8115 | 11185 | 4918 | 6687 | 8409 | 16672 | 12019 | 13799 | 8254 | 5305 | 11985 | 5812 | 11596 |
| **Nodes (n)** | 28 | 46 | 14 | 18 | 42 | 91 | 58 | 58 | 29 | 18 | 45 | 22 | 48 |
| **Boutons (n)** | 1758 | 2293 | 934 | 1344 | 1500 | 3526 | 2619 | 3084 | 1441 | 1171 | 2434 | 1023 | 2657 |
| **Bouton density** | 21.7 | 20.5 | 19.0 | 20.1 | 17.8 | 21.1 | 21.8 | 22.3 | 17.5 | 22.1 | 20.3 | 17.6 | 22.9 |
| **I hc** | 65 | 12 | 106 | 0 | 0 | 21 | 0 | 133 | 9 | 0 | 62 | 29 | 138 |
| **I sept** | 0 | 0 | 0 | 10 | 0 | 65 | 184 | 14 | 42 | 0 | 0 | 0 | 0 |
| **I nc** | 0 | 0 | 0 | 56 | 0 | 145 | 133 | 159 | 0 | 45 | 0 | 0 | 0 |
| **II hc** | 82 | 14 | 37 | 0 | 0 | 43 | 28 | 44 | 0 | 0 | 66 | 44 | 53 |
| **II sept** | 0 | 0 | 0 | 0 | 0 | 32 | 99 | 30 | 39 | 0 | 0 | 0 | 0 |
| **II nc** | 0 | 0 | 0 | 113 | 0 | 48 | 34 | 94 | 0 | 45 | 0 | 0 | 0 |
| **III hc** | 72 | 29 | 117 | 0 | 0 | 86 | 145 | 102 | 0 | 0 | 72 | 98 | 47 |
| **III sept** | 0 | 0 | 0 | 0 | 0 | 4 | 43 | 4 | 37 | 0 | 0 | 0 | 0 |
| **III nc** | 0 | 0 | 0 | 137 | 0 | 0 | 0 | 298 | 0 | 44 | 0 | 0 | 0 |
| **IV hc** | 66 | 71 | 62 | 0 | 0 | 220 | 129 | 109 | 0 | 0 | 111 | 58 | 81 |
| **IV sept** | 0 | 0 | 0 | 0 | 45 | 0 | 30 | 49 | 73 | 0 | 0 | 0 | 0 |
| **IV nc** | 36 | 0 | 0 | 166 | 11 | 0 | 0 | 222 | 35 | 70 | 0 | 0 | 0 |
| **Va hc** | 89 | 119 | 36 | 57 | 0 | 243 | 97 | 144 | 126 | 0 | 316 | 65 | 109 |
| **Va sept** | 9 | 56 | 17 | 12 | 20 | 0 | 4 | 47 | 66 | 0 | 28 | 51 | 42 |
| **Va nc** | 163 | 91 | 69 | 129 | 100 | 4 | 0 | 203 | 47 | 30 | 97 | 119 | 94 |
| **Vb hc** | 501 | 435 | 233 | 325 | 244 | 934 | 814 | 281 | 223 | 274 | 765 | 321 | 984 |
| **Vb sept** | 62 | 102 | 64 | 32 | 147 | 84 | 95 | 48 | 63 | 27 | 205 | 37 | 165 |
| **Vb nc** | 391 | 449 | 86 | 140 | 451 | 397 | 217 | 175 | 233 | 181 | 213 | 51 | 263 |
| **VI hc** | 8 | 275 | 6 | 147 | 77 | 141 | 294 | 262 | 133 | 106 | 163 | 32 | 289 |
| **VI sept** | 9 | 90 | 0 | 11 | 0 | 255 | 61 | 64 | 14 | 17 | 26 | 0 | 89 |
| **VI nc** | 204 | 422 | 0 | 0 | 0 | 629 | 108 | 280 | 52 | 130 | 77 | 0 | 243 |

**Supplementary Table 2b: Detailed axonal properties of all reconstructed RB layer Vb-pyramidal cells.**

All values are numbers (n) unless otherwise noted. Abbreviations: hc – home column; nc – neighboring column; sept – septum. Roman numerals name cortical layers.
