# Supplementary material for: Morphological Characteristics of Electrophysiologically Characterized Layer Vb Pyramidal Cells in Rat Barrel Cortex
Source: PLoS One. 2016 Oct 5;11(10):e0164004. doi: 10.1371/journal.pone.0164004 (PMC5051735; doi:10.1371/journal.pone.0164004)
Supplement: S3 Table — Mean ± standard deviation of the values plotted in Fig 3. (DOCX) [file pone.0164004.s006.docx]

|  | **RS** | **RB** | **RS** | **RB** |
| --- | --- | --- | --- | --- |
|  | basal dendrite [µm] | | apical dendrite [µm] | |
| home column | 2933.31 ± 868.08 | 5011.94 ± 1279.29 | 6564.31 ± 1717.52 | 7919.24 ± 1533.88 |
| extracolumnar | 111.25 ± 1.65.21 | 456.12 ± 621.62 | 105.38 ± 169.9 | 641.71 ± 890.07 |
|  |  |  |  |  |
| layer I |  |  | 1356.81 ± 668.40 | 2460.88 ± 1060.04 |
| layer II |  |  | 774.00 ± 393.25 | 1113.53 ± 743.70 |
| layer III |  |  | 355.93 ± 146.92 | 442.35 ± 207.11 |
| layer IV |  |  | 590.53 ± 240.15 | 600.65 ± 385.34 |
| layer Va | 57.81 ± 124.68 | 126.12 ± 241.37 | 1508.13 ± 789.60 | 2102.88 ± 934.23 |
| layer Vb | 2914.38 ± 782.05 | 5304.06 ± 1293.99 | 2121.19 ± 986.65 | 1840.65 ± 924.82 |
| layer VI | 72.38 ± 202.51 | 37.90 ± 74.89 |  |  |
|  |  |  |  |  |
| layer I home column |  |  | 1327.44 ± 658.55 | 2106.53 ± 861.70 |
| layer I extracolumnar |  |  | 29.38 ± 100.34 | 354.35 ± 393.26 |
| layer II home column |  |  | 774.00 ± 393.25 | 1113.53 ± 743.70 |
| layer Ii extracolumnar |  |  | 0 ± 0 | 0 ± 0 |
| layer III home column |  |  | 355.93 ± 146.92 | 442.35 ± 207.11 |
| layer III extracolumnar |  |  | 0 ± 0 | 0 ± 0 |
| layer IV home column |  |  | 584.87 ± 233.33 | 537.41 ± 231.71 |
| layer IV extracolumnar |  |  | 5.31 ± 21.25 | 63.24 ± 260.73 |
| layer Va home column | 57.81 ± 124.68 | 126.10 ± 241.37 | 1491.75 ± 762.26 | 1970.82 ± 863.61 |
| layer Va extracolumnar | 0 ± 0 | 0 ± 0 | 16.38 ± 48.50 | 132.06 ± 322.17 |
| layer Vb home column | 2803.13 ± 837.32 | 4847.94 ± 1260.71 | 2066.88 ± 952.15 | 1748.59 ± 875.90 |
| layer Vb extracolumnar | 111.25 ± 165.21 | 456.12 ± 621.62 | 54.31 ± 100.98 | 92.06 ± 140.61 |
| layer VI home column | 72.38 ± 202.51 | 37.90 ± 74.89 |  |  |
| layer VI extracolumnar | 0 ± 0 | 0 ± 0 |  |  |

**Supplementary Table 3:** Mean ± standard deviation of the values plotted in Fig. 3.
