# Supplementary material for: Morphological Characteristics of Electrophysiologically Characterized Layer Vb Pyramidal Cells in Rat Barrel Cortex
Source: PLoS One. 2016 Oct 5;11(10):e0164004. doi: 10.1371/journal.pone.0164004 (PMC5051735; doi:10.1371/journal.pone.0164004)
Supplement: S4 Table — Mean ± standard deviation of the values plotted in Fig 6. (DOCX) [file pone.0164004.s007.docx]

|  | **RS** | **RB** |
| --- | --- | --- |
|  | axonal boutons [n] | |
| home column | 2947.42 ± 1403.16 | 948.38 ± 510.71 |
| extracolumnar | 681.00 ± 501.50 | 872.92 ± 445.26 |
|  |  |  |
| layer I | 266.33 ± 418.74 | 109.85 ± 107.60 |
| layer II | 167.00 ± 85.18 | 72.69 ± 53.45 |
| layer III | 283.83 ± 138.09 | 102.69 ± 103.42 |
| layer IV | 402.58 ± 237.63 | 126.46 ± 90.82 |
| layer Va | 387.25 ± 177.99 | 223.00 ± 113.68 |
| layer Vb | 1313.33 ± 820.12 | 824.00 ± 381.29 |
| layer VI | 808.08 ± 552.77 | 362.62 ± 313.98 |
|  |  |  |
| layer I home column | 209.58 ± 371.94 | 44.23 ± 51.73 |
| layer I extracolumnar | 56.75 ± 101.51 | 65.62 ± 102.75 |
| layer II home column | 125.92 ± 88.39 | 31.62 ± 27.33 |
| layer Ii extracolumnar | 41.08 ± 57.58 | 41.08 ± 53.11 |
| layer III home column | 236.75 ± 139.00 | 59.08 ± 50.14 |
| layer III extracolumnar | 47.08 ± 40.98 | 43.62 ± 86.80 |
| layer IV home column | 337.00 ± 220.05 | 69.77 ± 63.98 |
| layer IV extracolumnar | 65.58 ± 90.82 | 56.69 ± 82.21 |
| layer Va home column | 345.75 ± 176.45 | 107.77 ± 89.76 |
| layer Va extracolumnar | 41.50 ± 37.24 | 115.23 ± 70.40 |
| layer Vb home column | 1052.67 ± 693.13 | 487.23 ± 284.14 |
| layer Vb extracolumnar | 260.67 ± 258.22 | 336.77 ± 163.07 |
| layer VI home column | 639.75 ± 452.03 | 148.69 ± 104.42 |
| layer VI extracolumnar | 168.33 ± 189.70 | 213.92 ± 256.23 |

**Supplementary Table 4:** Mean ± standard deviation of the values plotted in Fig. 6.
